# An Integrated Diagnosis Strategy for Congenital Myopathies

**DOI:** 10.1371/journal.pone.0067527

**Published:** 2013-06-24

**Authors:** Johann Böhm, Nasim Vasli, Edoardo Malfatti, Stéphanie Le Gras, Claire Feger, Bernard Jost, Nicole Monnier, Julie Brocard, Hatice Karasoy, Marion Gérard, Maggie C. Walter, Peter Reilich, Valérie Biancalana, Christine Kretz, Nadia Messaddeq, Isabelle Marty, Joël Lunardi, Norma B. Romero, Jocelyn Laporte

**Affiliations:** 1 Department of Translational Medicine and Neurogenetics, Institut de Génétique et de Biologie Moléculaire et Cellulaire, Illkirch, France; 2 Institut National de la Santé et de la Recherche Médicale, U964, Illkirch, France; 3 Centre national de la recherche scientifique, UMR7104, Illkirch, France; 4 Université de Strasbourg, Illkirch, France; 5 Collège de France, chaire de génétique humaine, Illkirch, France; 6 Institut de Myologie, Unité de Morphologie Neuromusculaire, GHU La Pitié-Salpêtrère, Paris, France; 7 Department of Neurological, Neurosurgical, and Behavioral Sciences, University of Siena, Italy; 8 Université Paris 6 UR76, Institut National de la Santé et de la Recherche Médicale UMR 974, Centre national de la recherche scientifique UMR 7215, Institut de Myologie, GHU La Pitié-Salpêtrière, Paris, France; 9 Centre de référence de pathologie neuromusculaire Paris-Est, Institut de Myologie, GHU La Pitié-Salpêtrière, Assistance Publique-Hôpitaux de Paris, Paris, France; 10 DNA microarrays and Sequencing platform, Institut de Génétique et de Biologie Moléculaire et Cellulaire, Illkirch, France; 11 Laboratoire de Biochimie Génétique et Moléculaire, CHU de Grenoble, Grenoble, France; 12 Institut des Neurosciences, Inserm U836, La Tronche, France; 13 Department of Neurology, Ege University School of Medicine, Izmir, Turkey; 14 Service de Génétique, CHU de Caen, Caen, France; 15 Friedrich-Baur-Institute, Department of Neurology, Ludwig-Maximilians University, Munich, Germany; 16 Faculté de Médecine, Laboratoire de Diagnostic Génétique, Nouvel Hopital Civil, Strasbourg, France; 17 Imaging Center, Institut de Génétique et de Biologie Moléculaire et Cellulaire, Illkirch, France; University Hospital Vall d'Hebron, Spain

## Abstract

Congenital myopathies are severe muscle disorders affecting adults as well as children in all populations. The diagnosis of congenital myopathies is constrained by strong clinical and genetic heterogeneity. Moreover, the majority of patients present with unspecific histological features, precluding purposive molecular diagnosis and demonstrating the need for an alternative and more efficient diagnostic approach. We used exome sequencing complemented by histological and ultrastructural analysis of muscle biopsies to identify the causative mutations in eight patients with clinically different skeletal muscle pathologies, ranging from a fatal neonatal myopathy to a mild and slowly progressive myopathy with adult onset. We identified *RYR1* (ryanodine receptor) mutations in six patients and *NEB* (nebulin) mutations in two patients. We found novel missense and nonsense mutations, unraveled small insertions/deletions and confirmed their impact on splicing and mRNA/protein stability. Histological and ultrastructural findings of the muscle biopsies of the patients validated the exome sequencing results. We provide the evidence that an integrated strategy combining exome sequencing with clinical and histopathological investigations overcomes the limitations of the individual approaches to allow a fast and efficient diagnosis, accelerating the patient’s access to a better healthcare and disease management. This is of particular interest for the diagnosis of congenital myopathies, which involve very large genes like *RYR1* and *NEB* as well as genetic and phenotypic heterogeneity.

## Introduction

Congenital myopathies (CM) are rare disorders characterized by early-onset muscle weakness and classified based on the predominance of particular histological anomalies on muscle biopsies. They have an estimated prevalence of about 1∶25 000 and are usually associated with neonatal or childhood onset, progressive or non-progressive muscle weakness, breathing difficulties and delayed motor milestones [1], [2]. The main congenital myopathy subgroups are protein aggregate myopathies (primarily nemaline myopathy), core myopathies and centronuclear myopathies (CNM), respectively characterized by rod-like protein accumulations, focal myofibrillar disorganization, and nuclear centralization on muscle biopsies [3]. Other congenital myopathy subgroups have been reported with different structural hallmarks [4]. As congenital myopathies are usually severe with a high recurrence risk in affected families, molecular diagnosis is important to provide an adequate healthcare and genetic counseling.

Although many genes have been associated with congenital myopathies in the past years, a recent study reported that only 16 out of 46 US patients were molecularly diagnosed [5]. This is due to the fact that despite clinical and histological examinations, the majority of the patients presented with unspecific features. Especially for the neonatal cases, a reliable diagnosis is often challenging. Another reason is the genetic heterogeneity in congenital myopathies with the implication of more than 20 known genes [4], opposing efficient molecular diagnosis. In addition, some of the genes implicated in congenital myopathies belong to the largest genes of the human genome, as *TTN* (363 exons; MIM#188840) mutated in congenital myopathy with fatal cardiomyopathy, *NEB* (183 exons; MIM#161650) mutated in nemaline myopathy, or *RYR1* (106 exons; MIM#180901) mutated in different pathologies. The aim of this study was to propose and validate an integrated approach including exome sequencing for the diagnosis of congenital myopathies with neonatal and adult onset. The next generation sequencing technology has become an effective strategy for massively parallel analysis of a large number of genes and has led to the successful identification of several Mendelian disease genes [6]. This approach is however uncommonly used in routine molecular diagnosis despite its potential synergy with clinical and histological investigations. Sanger sequencing of single genes remains the major technique for monogenetic pathologies with characteristic clinical manifestations. It is time-consuming and not centralized, demonstrating the need for a more efficient diagnostic approach.

Here we used an integrated exome sequencing strategy to identify the causative mutations in eight patients from six families with clinically different neonatal or adult-onset congenital myopathies. We found pathogenic mutations in the large *RYR1* and *NEB* genes, and histopathological and ultrastructural analysis of the muscle biopsies of the patients confirmed and validated the exome sequencing results. In conclusion, we provide the evidence that exome sequencing in combination with histological analyses is a fast, efficient and reliable method to identify disease-causing mutations in unsolved myopathy cases. Our integrated approach is particularly relevant for disease groups with genetic and phenotypic heterogeneity.

## Materials and Methods

### Patients

Patients originated from France (Families 1 and 2), Greece/Morocco (Family 3), French West Indies (Family 4), Germany (Family 5), and Turkey (Family 6). Sample collection was performed with written informed consent from the patients or their legal guardians according to the declaration of Helsinki and experimentation was approved by the INSERM institutional review board (“Comité de protection des personnes Est IV”). Muscle biopsies were obtained from deltoid (ARX30, AKY21, IM26, AHY58, AHE6), biceps brachii (ATG66, AGT67) or tibialis anterior ARX33).

### Linkage analysis

For whole-genome analysis, the genomic DNA of patient AHE6 was hybridized on Affymetrix SNP array 6.0 according to the manufacturer's instructions. Loss of heterozygosity was analyzed with GeneChip DNA Analysis and Chromosome Copy Number Analysis softwares (Affymetrix, Santa Clara, CA, USA).

### Exome sequencing

Genomic DNA was prepared from peripheral blood by routine procedures and quality-controlled. DNA was sheared using the Covaris E210 (KBioscience, Herts, UK) followed by automatic library preparation with the SPRI-TE machine (Beckman Coulter Inc., Brea, CA, USA). Exon capture was performed with the Agilent SureSelect Array v1 (families 3–6) or the refined Agilent SureSelect Human all Exon 50 Mb Kit (targeting all NEB and TTN exons; Families 1 and 2) (Agilent Technologies, Santa Clara, CA, USA). Enriched DNA fragments were sequenced on an Illumina Genome Analyzer IIx to generate 72nt single reads for AHE6 and paired-end reads for AGT66, AGT67, AHY58, AKY21 and IM26. For patients ARX30 and ARX33 we performed “trio sequencing”, i.e. exome sequencing with 72 nt paired-end reads of the patient and both healthy parents.

### Bioinformatic analysis

Sequence data were analyzed using Illumina Pipeline RTA (Real-Time Analysis) version 1.7 and aligned to the reference genome GRCh37/hg19 using BWA [7]. Variant calling and filtering of reads sharing the same start position and strand was done with Samtools [8]. Variants were considered as heterozygous when present in 20 – 80 % of the reads and as homozygous when present in ≥ 80 % of the reads. For SNP/indel annotation and filtering SVA, Ensembl60, dbSNP134, 1000 genomes, and NHLBI exome variant server were used. Impact of variations were predicted using SIFT [9] (maximum pathogenic score: 0), PolyPhen V2 [10] (maximum pathogenic score: 1), NNSPLICE [11] and Human Splicing Finder [12] (Table S5).

### Mutation characterization

Mutation confirmation and segregation analysis were performed by PCR and Sanger sequencing of the *RYR1*/*NEB* exons harboring the mutations and the adjacent exon-intron boundaries. Primer sequences and PCR conditions are listed in Table S2. The mutations were numbered according to GenBank NM_000540.2 and NP_000531.2 (*RYR1*) and NM_001164507.1 and NP_001157979.1 (*NEB*). Nucleotide position reflects cDNA numbering with +1 corresponding to the A of the ATG translation initiation codon.

### Muscle histology

For histochemical analyses, transverse sections (10 µm) of the muscle biopsies were stained with hematoxylin-eosin, Gomori trichrome, NADH tetrazolium reductase (NADH-TR), periodic acid-Schiff and ATPase and assessed for nuclei position, fiber morphology, fiber type distribution, cores and accumulations/infiltrations.

### Electron microscopy

Muscle sections were fixed in 2.5% paraformaldehyde, 2.5% glutaraldehyde, and 50 mM CaCl_2_ in 0.1 M cacodylate buffer (pH 7.4), and post-fixed with 2% OsO_4_, 0.8% K_3_Fe(CN)_6_ in 0.1 M cacodylate buffer (pH 7.4) for 2 h at 4°C and incubated with 5% uranyl acetate for 2 h at 4°C. Samples were dehydrated in graded series of ethanol and embedded in epon resin 812. Ultrathin sections (70 nm) were contrasted with uranyl and lead citrate and viewed at 70 kv with a Morgagni 268D electron microscope and a Mega View III camera (Soft Imaging System, Münster, Germany).

### RNA

RNA was extracted from the deltoid muscle biopsy of patients ARX30, IM26 and AHE6 and from a tibialis anterior biopsy of patient ARX33 using Tri reagent (Molecular Research Center Inc., Cincinnati, OH, USA), and reverse transcribed using the SuperScript® III kit (Invitrogen, Carlsbad, CA, USA). Sequencing of the entire *RYR1* cDNA of IM26 and AHE6 confirmed the presence of the *RYR1* mutations identified by exome sequencing and excluded further sequence aberrations. The impact of the *NEB* mutations on splicing in patients ARX30 and ARX33 was assessed using *NEB* specific primers.

### Protein

Western blot was performed using routine protocols on a deltoid muscle biopsies from AHE6 and a healthy age-matched control using a home-made rabbit anti-RYR1 [13] and a DE-R-11 mouse anti-Desmin antibody (Dakocytomation, Trappes, France) for normalization. Signals were detected with a chemiluminescent HRP substrate and quantified using a ChemiDoc XRS apparatus (Biorad, Marnes la Coquette, France) and the Quantity One software (Biorad). The quantification was repeated 6 times.

## Results

### An integrated diagnosis approach for congenital myopathies

We studied eight patients from six families with different clinical and histological features suggestive of congenital myopathies. The neonatal forms ranged from fatal shortly after birth to moderately progressive, and the adult form was mild and slowly progressive (Table 1). Using Sanger sequencing, the patients were previously excluded for several genes implicated in nemaline or centronuclear myopathies, including *ACTA1*, *TPM2*, *TPM3*, *TNNT1* for families 1 and 2, *MTM1* and *BIN1* for families 3 to 6, and *DNM2* for families 3, 4 and 6. These inconclusive preliminary analyses underscore the necessity of an alternative and more efficient molecular diagnosis. We therefore used an integrated approach, combining exome sequencing and histological investigations. Exome sequencing was performed for all patients and for the parents of families 1 and 2 (trio sequencing) and pointed - in combination with histopathology - unambiguously to single myopathy genes. A statistical overview of the sequencing results is shown in Tables 2, 3, 4, 5 and the coverage of each exon of the known genes implicated in congenital myopathies is specified in Tables S3 and S4. The inheritance pattern was taken into account for the selection of candidate genes. For the sporadic cases in families 1 and 2, genes with two heterozygous variants segregating from each parent were selected as the analysis for de novo mutations was not conclusive. For the non-consanguineous families 3 and 5 with two affected members each, genes with two common heterozygous variations were selected. For family 4, we verified dominant and recessive scenarios. For patient AHE6 from the consanguineous family 6, we focused on homozygous variants mapped in large homozygous regions, determined by homozygosity-by-descent (Table S1). The impact of potential mutations was predicted with SIFT and PolyPhen for amino acid changes and with NNSPLICE and Human Splicing Finder for changes potentially affecting splicing. All identified mutations were verified by Sanger sequencing in the starting genomic DNA and also in the cDNA for families 1, 2, 3 and 6. Histological analysis of muscle biopsies was performed for all patients. For five patients (ARX30, AKY21, IM26, AHY58 and AHE6), we additionally analyzed the muscle biopsy by electron microscopy.

**Table 1:** Phenotypic and molecular data.

	Family 1	Family 2	Family 3		Family 4	Family 5		Family 6
Patient	ARX30	ARX33	AKY21	IM26	AHY58	AGT66	AGT67	AHE6
Disease								
occuren	Sporadic	Sporadic	2 affected children		Sporadic	2 affected children		Sporadic (consanuineous)
ce								
Origin	France	France	Greece/Morocco		French West Indies	Germany		Turkey
Myopath y	Severe with arthrogryposis	Severe	Severe		Severe	Moderately progressive		Slowly progressive
Onset	Neonatal	Neonatal	Neonatal		Neonatal	Neonatal		Adulthood
Age at								
last examina	Deceased at 10 days	Deceased at 2 days	Deceased at 45 days	Deceased at 7 days	1	45	31	35
tion								
Motor function	Generalized hypotonia	Generalized hypotonia	Generalized hypotonia	Generalized hypotonia	Mild muscle weakness of upper limbs, walking difficulties	Diffuse muscle weakness, wheelchair-bound	Diffuse muscle weakness, walks short distances	Mild muscle weakness of upper limbs, walking difficulties
Respirati on	Abnormal	Abnormal	Abnormal	Abnormal	Abnormal	Normal	Normal	Normal
Facial involvem ent	n.d.	n.d.	n.d.	n.d.	Facial weakness	Facial weakness, ptosis	Facial weakness, ptosis	No
Histopat hological hallmark s	Nemaline bodies, fiber size variability, type I fiber predominance	Nemaline bodies	Internal nuclei, fiber size variability, atrophy, central cores	Internal nuclei, fiber size variability, atrophy, central cores	Internal nuclei, fiber size variability, atrophy,cores	Internal nuclei, type I fiber predominance, fiber size variability, multi minicores	Internal nuclei, type I fiber predominance, fiber size variability, multi minicores	Internal nuclei, type I fiber predominance, fiber size variability, radial arrangements of sarcoplasmic strands, necklace fibers, central cores
Gene	NEB	NEB	RYR1	RYR1	RYR1	RYR1	RYR1	RYR1
Protein	Nebulin	Nebulin	Ryanodine receptor 1		Ryanodine receptor 1	Ryanodine receptor 1		Ryanodine receptor 1
Mutation	**c.5574C>G**; **c.19101+5G>A**	**c.5783_5784delAT**; **c.8160+1G>A**	**c.3223C>T**; c.7025A>G; (**c.7645-7650dupGCGCTG**)		**c.8953C>T**; **c.9758T>C**	c.325C>T; **c.8140_8141delTA**		**c.8888T>C**, homozygous
Predicte d protein impact	p.Tyr1858*; **p.Leu6333_**	p.Tyr1928fs*2; **p.Asn2653_His2720del**	**p.Arg1075Trp**; p.Asn2342Ser; (**p.Ala2549_Leu2550dup**)		p.Arg2985*; **p.Ile3253Thr**	p.Arg109Trp; p.Tyr2714fs*7		**p.Leu2963Pro**

n.d.  =  not determined.

Bold  =  new mutations.

For family 3, the (c.7645-7650dupGCGCTG) change is a potential modifier of the phenotype.

**Table 2:** Statistical overview of the exome sequencing results: sporadic cases (trio sequencing) ARX 30 and ARX33.

Sporadic cases (trio sequencing); paired-end 72nt	ARX30	ARX33
Mean coverage	56	55
Coverage nt ≥ 10x	86 %	87 %
Total SNVs	43 062	43 733
SNVs spice sites	87	41
SNV nonsense	10	12
SNV missense	563	445
Total indels	6245	5838
Indels frameshift	595	329
Heterozygous SNVs splice shared with one parent	12	11
Heterozygous SNVs nonsense shared with one parent	3	2
Heterozygous SNVs missense shared with one parent	75	89
Heterozygous indels frameshift shared with one parent	17	16
Two het. SNVs/indels in same gene from distinct parents	**2**	**1**
**Myopathy gene**	**1 (NEB)**	**1 (NEB)**

**Table 3:** Statistical overview of the exome sequencing results: familial cases (non-consanguineous) AKY21/IM26 and AGT66/AGT67.

Familial cases (non-consanguineous); paired-end 72 nt	AKY21	IM26	AGT66	AGT67
Mean coverage	59	73	63	63
Coverage nt ≥ 10x	90.0 %	90.7 %	90.2%	90.1 %
Total SNVs	26 897	29 011	27 273	26 815
SNVs spice sites	164	174	138	110
SNV nonsense	24	16	15	9
SNV missense	3 573	4 083	3 685	2 623
Total indels	1 838	2 048	1 864	1 864
Indels frameshift	51	69	50	51
Common heterozygous SNVs splice	3		2	
Common heterozygous SNVs nonsense	8		5	
Common heterozygous SNVs missense	385		306	
Common heterozygous indels frameshift	15		12	
Two Common het. SNVs/indels in same gene	**39**		**25**	
**Myopathy gene**	**1 (RYR1)**		**1 (RYR1)**	

**Table 4:** Statistical overview of the exome sequencing results: sporadic case AHY58.

Sporadic case; paired-end 72nt	AHY58
Mean coverage	59
Coverage nt ≥ 10x	94%
Total SNVs	29182
SNVs spice sites	103
SNV nonsense	167
SNV missense	9480
Total indels	2381
Indels frameshift	155
Two heterozygous SNVs/indels in same gene	20
**Myopathy gene**	**1 (RYR1)**

**Table 5:** Statistical overview of the exome sequencing results: sporadic case (consanguineous) AHE6.

Sporadic case (consanguineous); single read 72nt	AHE6
Mean coverage	23
Coverage nt ≥ 10x	75%
Total SNVs	35755
SNVs spice sites	238
SNV nonsense	31
SNV missense	4756
Total indels	3393
Indels frameshift	256
Linked homozygous SNVs splice	0
Linked homozygous SNVs nonsense	0
Linked homozygous SNVs missense	18
Linked homozygous indels frameshift	0
Total linked homozygous SNVs/indels	**18**
**Myopathy gene**	**1 (RYR1)**

### Clinical and histological findings and mutation detection by exome sequencing

**Figure 1:**
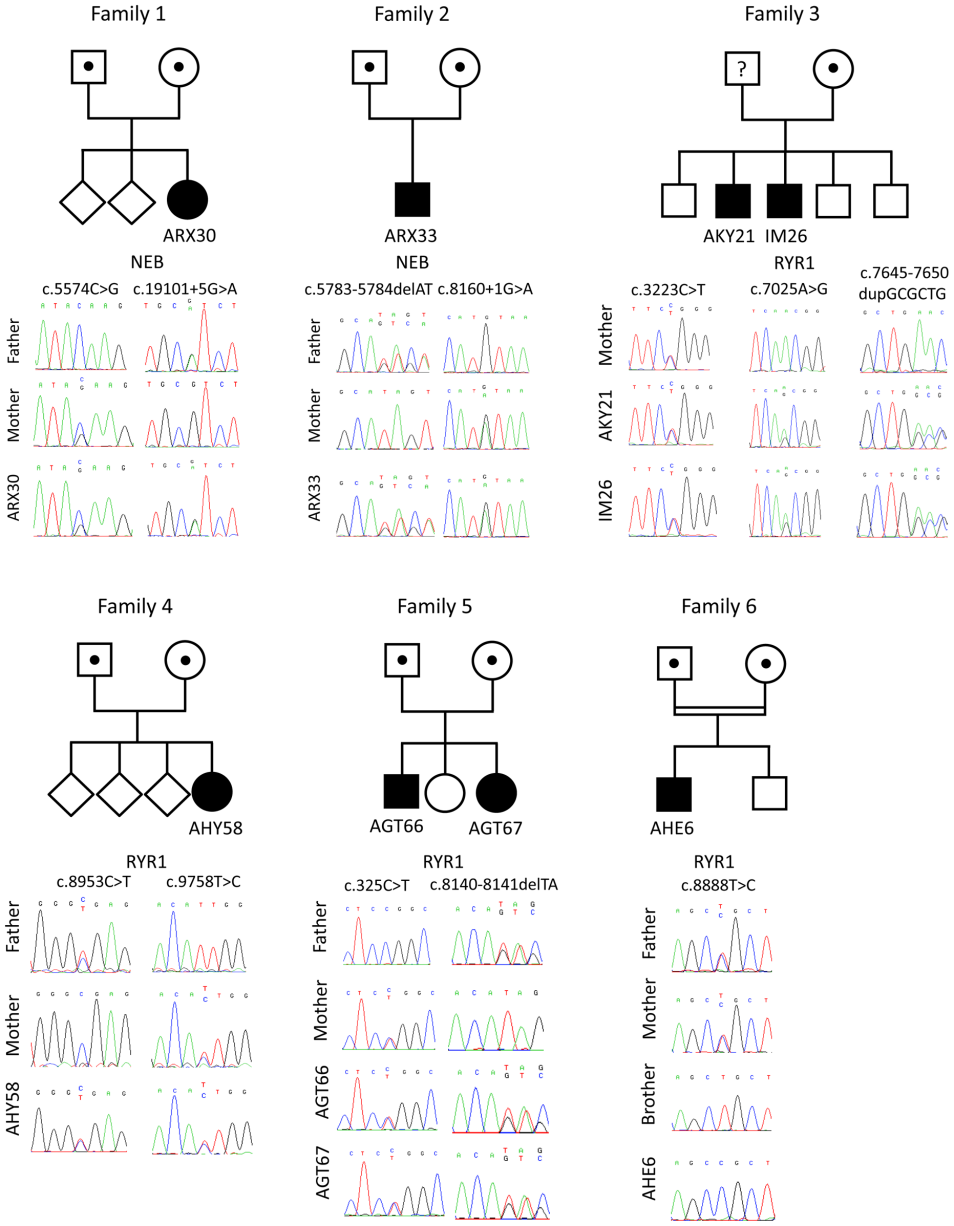
Pedigrees and*RYR1/NEB* mutations in six families with different muscle disorders. Patient ARX30 harbors the *NEB* c.5574C>G mutation on the maternal and the *NEB* c.19101+5G>A mutation on the paternal allele. Patient ARX33 harbors the *NEB* c.8160+1G>A mutation on the maternal and the *NEB* c.5783_5784delAT mutation on the paternal allele. Patients AKY21 and IM26 carry the heterozygous c.3223C>T, c.7025A>G and c.7645-7650dupGCGCTG mutations in *RYR1*. The c.3223C>T mutation was found on the maternal allele, the father’s DNA was not available. AHY58 harbors two heterozygous *RYR1* mutations: c.8953C>T on the paternal allele and c.9758T>C on the maternal allele. In patients AGT66 and AGT67 we identified the heterozygous *RYR1* mutations c.325C>T on the maternal and c.8140_8141delTA on the paternal allele. Patient AHE6 from a consanguineous family was found to harbor the homozygous *RYR1* c.8888T>C mutation, both parents were heterozygous.

**Figure 2:**
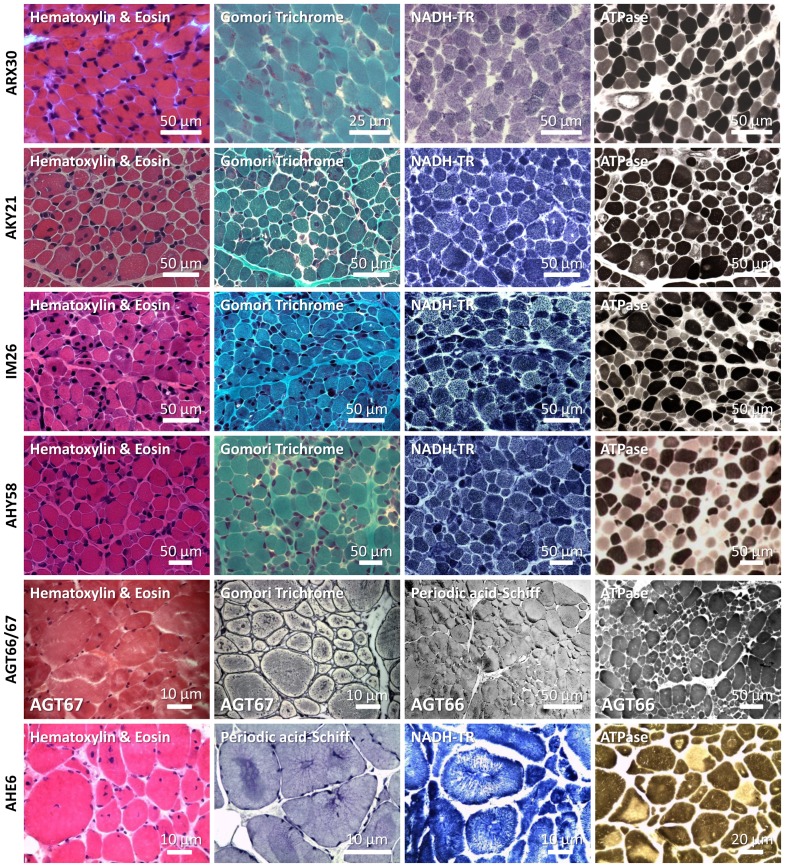
Histological analysis of muscle biopsies from patients ARX30, AKY21, IM26, AHY58, AGT66/AGT67, and AHE6. The deltoid muscle biopsy of patient ARX30 was performed 2 days after birth and revealed nemaline bodies, fiber size variability and type I fiber predominance. On the deltoid muscle biopsies from AKY21 and IM26 (shortly after birth), nuclear internalization, atrophy, fiber size variability, and areas devoid of oxidative enzyme activity became apparent. Analysis of the deltoid muscle biopsy of patient AHY58 (20 days) demonstrated fiber size variability, atrophy, internal nuclei, and discrete areas of reduced oxidative enzyme activity. Biceps brachii biopsy from AGT66 (8 years) and AGT67 (shortly after birth) revealed nuclear internalization, fiber size variability, multiple minicores devoid of oxidative enzyme activity and type I fiber predominance. Left deltoid muscle biopsy from patient AHE6 (performed at 30 years) revealed internalized nuclei, fiber size variation, radial arrangements of sarcoplasmic strands, necklace fibers, type I fiber predominance, core-like structures, fibrosis and fatty infiltrations.

**Figure 3:**
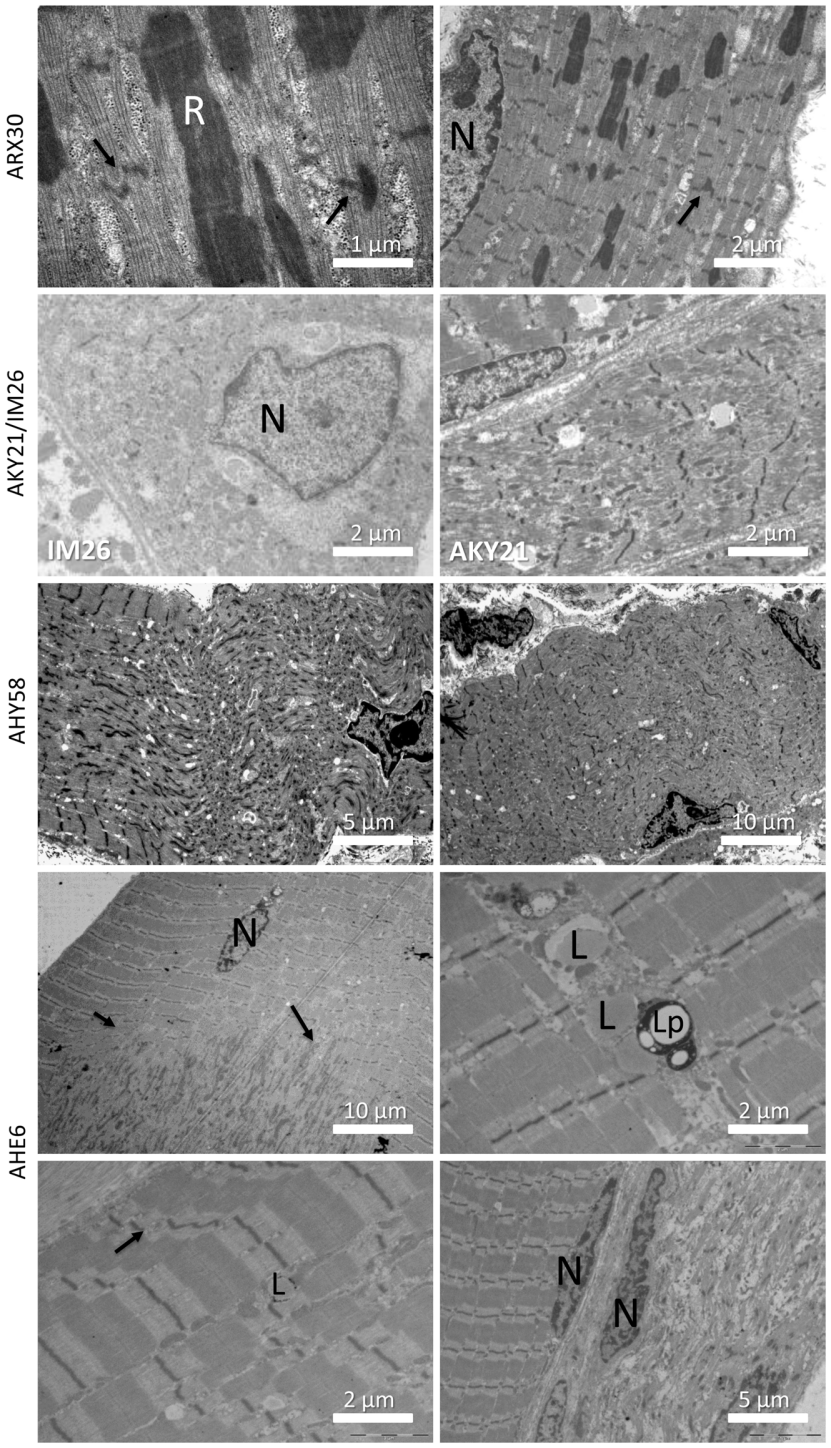
Ultrastructural analysis of muscle biopsies. Ultrastructural analysis of the biopsy from patient ARX30 revealed myofibrillar disorganization, Z-line streaming of adjacent sarcomeres (arrows), and prominent nemaline rods. The biopsy of patient IM26 showed large disorganized areas around internalized nuclei, the longitudinal muscle section of AKY21 revealed prominent Z-band streaming. Analysis of the biopsy of AHY58 demonstrated marked myofibrillar disorganization, fragmented Z-bands and internalized nuclei. The biopsy of patient AHE6 displayed nuclear centralization, myofibrillar disorganization, fibrosis, lipofuscin granules and myofiber degeneration. R  =  nemaline rods, N  =  nuclei, L  =  lipids, Lp  =  lipofuscin

**Figure 4:**
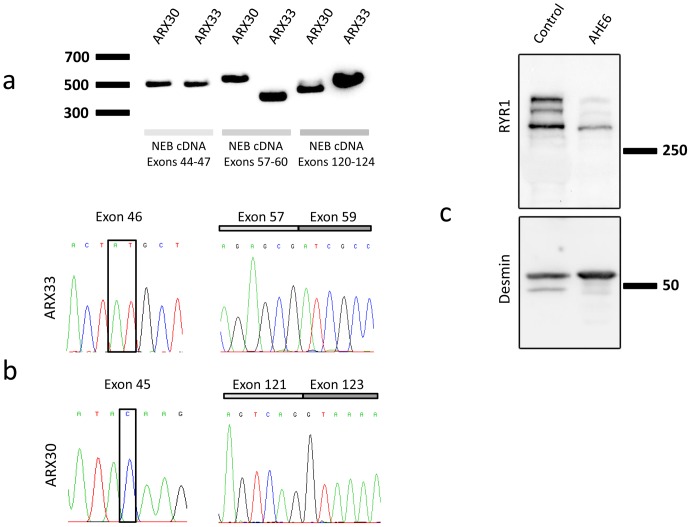
Impact of the*NEB/RYR1* mutations. (A) Normal sized cDNA amplicons for exons 44 to 47 from patient ARX30 with the heterozygous c.5574C>G nonsense mutation (exon 45) and from patient ARX33 with the heterozygous c.5783_5784delAT deletion (exon 46). The splice mutation c.8160+1G>A (intron 58, ARX33) resulted in a shorter *NEB* cDNA amplicon (exons 57–60) compared to the control. The splice mutation c.19101+5G>A (intron 122, ARX30) involved a weak cDNA amplicon (exons 120–124) of normal size and a strong amplicon of smaller size. (B) The *NEB* c.8160+1G>A splice mutation (ARX33) causes a complete skipping of the in-frame exon 58. The c.5783_5784delAT mutation was not seen in the cDNA, indicating mRNA degradation by nonsense-mediated mRNA decay (NMD) of the allele containing this deletion. The *NEB* cDNA amplicon of patient ARX30 (exons 122–126) did not contain the in-frame exon 122. The c.5574C>G mutation in exon 46 was not seen by cDNA sequencing, suggesting NMD of the allele harboring this nonsense mutation. (C) Western blot of a deltoid muscle extract revealed a strong reduction of the RYR1 protein level in patient AHE6 compared to a healthy age-matched control. Desmin was used for normalization.

For patient ARX30 from family 1, polyhydramnios and fetal akinesia were diagnosed during pregnancy and the patient presented with severe neonatal hypotonia, respiratory distress, arthrogryposis, hip hyperlaxity, club feet and dysmorphic features. Through exome sequencing, we identified two heterozygous mutations in *NEB*: the c.5574C>G (exon 45; p.Tyr1858*) nonsense mutation on the maternal allele and the c.19101+5G>A (intron 122) mutation on the paternal allele, confirming autosomal recessive inheritance (Figure 1). A deltoid muscle biopsy revealed nemaline rods, marked fiber size variability and type I fiber predominance (Figure 2). Ultrastructural analysis confirmed the presence of numerous nemaline bodies, and revealed mild disorganization of the myofibrillar structure and Z-band streaming (Figure 3). These findings were consistent with the mutations in *NEB* found by exome sequencing. We performed additional molecular analysis to confirm the impact of the mutations. The paternal mutation was predicted to impair splicing of exon 122 and RNA reverse transcription and cDNA sequencing confirmed major skipping of this in-frame exon (Figure 4). The nonsense mutation was not seen on the cDNA sequence, suggesting degradation of the maternal allele by nonsense-mediated mRNA decay (NMD).

Patient ARX33 from family 2 presented with a severe muscle weakness at birth and deceased in the following days due to cardio-respiratory arrest. We identified one *NEB* mutation on the paternal allele (c.5783_5784delAT) in exon 46, involving a frameshift and a premature stop codon (p.Tyr1928fs*2), and one *NEB* donor splice site mutation on the maternal allele (c.8160+1G>A, intron 58) (Figure 1). Gomori trichrome staining of a tibialis anterior muscle biopsy revealed nemaline rods (Figure S1), electron microscopy data were not available. The impact of the splice mutation was verified by muscle mRNA analysis and demonstrated complete skipping of the in-frame exon 58. The 2 nucleotides deletion was not seen on the cDNA sequence, suggesting nonsense-mediated mRNA decay of the paternal allele (Figure 4).

AKY21 and IM26 from family 3 deceased from a severe muscle disorder shortly after birth. Pregnancy was normal and both had low Apgar scores, generalized hypotonia and respiratory distress at birth. Cardiac examinations were normal. We detected three heterozygous *RYR1* mutations in exons 25 (c.3223C>T; p.Arg1075Trp), 43 (c.7025A>G; p.Asn2342Ser), and 48 (c.7645_7650dupGCGCTG; p.Ala2549_Leu2550dup). A DNA sample of the father was not available and sequencing of the maternal DNA revealed the presence of the c.3223C>T mutation in the absence of the other sequence aberrations, suggesting autosomal recessive inheritance with compound heterozygosity (Figure 1). The c.3223C>T (p.Arg1075Trp) missense mutation on the maternal allele affects a conserved amino acid (Figure S2) and is predicted to be highly damaging by Polyphen (score 1.000) and SIFT (Score 0.000). The c.7025A>G (p.Asn2342Ser) missense on the paternal allele has been associated with malignant hyperthermia susceptibility [14] (rs147213895), known to result from heterozygous *RYR1* mutations. Both missense mutations were found with equal intensity in the cDNA obtained by reverse transcription of the RNA from the patient muscle biopsy, suggesting that both alleles are equally expressed and that the mutations do not impact on mRNA stability. The deltoid muscle biopsies of both patients revealed nuclear internalization, atrophy, fiber size variability, and areas devoid of oxidative enzyme activity (Figure 2). Ultrastructural analysis of the biopsy of the patients showed that the disorganized myofibrillar areas around the internalized nuclei cover a large part of the fiber diameter. The longitudinal muscle section revealed prominent Z-band streaming (Figure 3). These findings were suggestive of a myopathy with cores. As *RYR1* mutations mostly involve cores, the histopathological findings and the exome sequencing results are in agreement.

AHY58 from family 4 was born with general amyotrophy, axial and peripheral hypotonia and arthrogryposis after normal pregnancy. Although the health status slightly improved, the patient had temporal respiratory distress and deglutition problems. We identified one heterozygous *RYR1* mutation on each parental allele, confirming autosomal recessive inheritance (Figure 1). The paternal c.8953C>T (p.Arg2985*) mutation in exon 59 creates a premature stop codon and the maternal c.9758T>C (p.Ile3253Thr) involves a missense substitution in exon 66, is highly conserved across species (Figure S2) and is predicted to be deleterious by Polyphen (0.981) and SIFT (0.001). None of these mutations were listed in the SNP databases. The deltoid muscle biopsy revealed nuclear internalization, atrophy, fiber size variability, and areas devoid of oxidative enzyme activity corresponding to cores (Figure 2). Ultrastructural analysis revealed atrophy, myofibrillar disarray and morphological alterations of the Z-line (Figure 3). Previous studies reported *RYR1* mutations associated with prominent nuclear internalization and areas of myofibrillar disorganization [15], [16] as for AHY58. The *RYR1* mutations identified by exome sequencing are therefore likely to be disease-causing.

AGT66 and AGT67 from family 5 are now 45 and 31 years old and presented with neonatal hypotonia without need for respiratory support. Motor milestones were delayed. Currently, AGT66 is wheelchair-bound and AGT67 is able to walk short distances. For both patients, diffuse muscle weakness, facial weakness and ptosis were diagnosed. They have contractures and absent reflexes. By comparing the exome sequencing data of both patients, we identified common compound heterozygous *RYR1* mutations, confirming autosomal recessive inheritance (Figure 1). The c.325C>T (p.Arg109Trp) missense mutation in exon 4 on the maternal allele has been reported to be associated (in combination with a second *RYR1* mutation) with multi-minicore and central core disease [17], [18]. The c.8140_8141delTA deletion in exon 51 on the paternal allele has never been described and is predicted to induce a frameshift and a premature TAA stop codon (p.Tyr2714fs*7). The biceps brachii biopsies of patients AGT66 and AGT67 revealed nuclear internalization, fiber size variability, type I fiber predominance, and multiple dot-like areas devoid of oxidative enzyme activity, suggestive of multiminicore disease (MmD) (Figure 2). MmD is a recessive disorder linked to mutations in *RYR1*, the exome sequencing results are therefore in agreement with the histological data.

Patient AHE6 from family 6 is a 35 year old male from a consanguineous family. Pregnancy and motor development were normal. At the age of 30 he started to feel increased fatigability, had walking difficulties and impaired manual capacities. Calf muscles were hypertrophic and mild muscle weakness was only present in the upper limbs. Except for the ankles, deep tendon reflexes were absent. CPK levels were elevated. We identified a homozygous *RYR1* missense mutation in exon 58 (c.8888T>C; p.Leu2963Pro) in a homozygous 21.1 Mb region on chromosome 19 encompassing the entire *RYR1* gene (Table S1), affecting a highly conserved amino acid (Figure S2) and predicted to be highly deleterious by Polyphen (0.999) and SIFT (0.000). Both parents were found to be heterozygous for the mutation, confirming autosomal recessive inheritance (Figure 1). The mutation was not listed in the SNP databases and was not found in 80 Turkish control chromosomes. The deltoid muscle biopsy of AHE6 revealed type I fiber predominance and core-like structures, suggestive of a core myopathy in agreement with the exome sequencing results (Figure 2). In addition, aberrant nuclear positioning, marked fiber size variation fibrosis and fatty infiltrations were observed. The radial arrangements of sarcoplasmic strands (“spoke of a wheel”) and necklace fibers are however untypical for core myopathies and are rather seen in centronuclear myopathy. However, mutations in *MTM1*, *BIN1* and *DNM2* were excluded by Sanger sequencing. Major findings by electron microscopy were nuclear internalization, fibrosis, massive fatty infiltrations, myofibrillar disorganization, myofiber degeneration, and lipofuscin granules (Figure 3). As the c.8888T>C; p.Leu2963Pro mutation was not previously reported, we analyzed a deltoid muscle extract by Western blot and found a strong decrease of the RYR1 protein level (down to 37%) compared to an age-matched control (Figure 4c). Summing up the histological and ultrastructural findings as well as the linkage analysis, exome sequencing and Western blot results, the *RYR1* p.Leu2963Pro missense is most probably the disease-causing mutation.

## Discussion

This study provides the evidence that exome sequencing – in combination with clinical and histological analyses - is a fast, efficient, and reliable molecular diagnosis tool for congenital myopathies. For consanguineous families, complementary homozygosity mapping can help to refine the chromosomal regions potentially harboring the disease-causing variants and thereby prioritize individual variations identified by exome sequencing. Using this integrated diagnosis strategy, we identified the pathogenic mutations in eight patients from six families with different muscle phenotypes, including a fatal muscle disorder with neonatal onset, a severe myopathy with neonatal onset, a slowly progressive form with neonatal onset and a mild and slowly progressive myopathy with adult onset.

### Mutations in*NEB* and *RYR1*

Patients from families 1 and 2 died from a severe myopathy shortly after birth, and both were found to harbour *NEB* mutations. *NEB* codes for the contractile unit scaffold protein nebulin and mutations in *NEB* usually give rise to a mild childhood or adult onset myopathy, but also rare fatal cases as for our patients have been reported [19], [20], [21]. The patients from families 3, 4, 5 and 6 were all found to carry mutations in *RYR1*, although they were clinically different. The patients from family 3 had a severe neonatal myopathy and deceased within a few days after birth. The patient from family 4 had a severe, but not fatal neonatal myopathy. Both patients from family 5 presented with a slowly progressive diffuse myopathy at birth and the patient from family 6 had a mild and adult-onset muscle weakness. These findings emphasize the phenotypic variability of *RYR1*–related disorders. Mutations in *RYR1* have been associated with different neuromuscular phenotypes including central core disease (CCD, MIM# 117000) [22], [23], multiminicore disease (MmD, MIM# 255320) [24], congenital myopathy with central or internalized nuclei [15], [16], congenital fiber-type disproportion (CFTD, MIM# 255310) [25], foetal akinesia [26], benign Samaritan congenital myopathy [27], and malignant hyperthermia susceptibility (MH, MIM# 145600) [28]. *RYR1* codes for the skeletal muscle ryanodine receptor, a key player in skeletal muscle excitation-contraction coupling. The majority of mutations in *RYR1* are missense mutations and most are associated with MH and CCD. Dominant CCD-related *RYR1* mutations predominantly occurred in the C-terminal hydrophobic domain, while recessive mutations giving rise to a wide range of clinico-pathological phenotypes were detected along the entire gene [29]. In this study we identified 5 missense mutations in the *RYR1* exons 4, 25, 43, 58 and 66 and confirm that there is no obvious hotspot or genotype-phenotype correlation for recessive mutations.

### Advantages of the integrated diagnosis strategy

The integrated diagnosis strategy combining exome sequencing with clinical and histological investigations is suitable for congenital myopathies for several reasons. First, congenital myopathies are clinically heterogeneous and our and other studies have demonstrated that especially the neonatal forms are often not clinically consistent, so that several candidate genes can be considered. Second, congenital myopathies are also genetically heterogeneous and mutations in several large genes account for a large number of cases. Protein aggregate myopathy for instance is linked to mutations in 7 different genes to date (*ACTA1*, *TPM2*, *TPM3*, *TNN1*, *NEB*, *CFL2*, *KBTBD13*). Third, congenital myopathies are classified based on histological hallmarks, but the majority of patients present non-specific features [5]. Some patient biopsies display a mix of several histological hallmarks as it is the case for our patient from family 6, increasing the number of candidate genes. Forth, the analysis can be performed in a unique laboratory, while Sanger sequencing is mostly performed in different diagnostic centers specialized for single or a small number of genes.

### Exome sequencing and histological analyses are complementary

Massively parallel sequencing allows a fast testing of all genes previously linked to a given disease, including the large genes [30]. Importantly, exome sequencing also covers new myopathy genes that will be discovered in the future. The dropping costs of this technique pave the way for a routine use in molecular diagnosis and are already far below the estimated expenses for classical Sanger sequencing of all exons of large genes as *RYR1* or *NEB*. However, exome sequencing does not detect intronic mutations and may generate a large list of variants of uncertain significance. The validation of the disease-causing mutations therefore requires the synergistic combination of the exome sequencing data with clinical and histological analyses. Exome sequencing and histology can be performed in parallel and the results need to be evaluated by specialized diagnostic centers and associated research laboratories with an expertise on the underlying pathophysiological mechanisms.

## Conclusion

Exome sequencing or targeted massively parallel sequencing improves molecular diagnosis of myopathies when combined with histopathology and molecular validation, and can accelerate the patient’s access to a better healthcare and disease management.

## Supporting Information

Figure S1 **Gomori trichrome staining of a tibialis anterior muscle section from patient ARX33 revealed the presence of nemaline rods.**
(TIF)

Figure S2 **The novel** ***RYR1*** **mutations c.3223C>T (Family 3), c.8888T>C (Family 6) and c.9758T>C (Family 5) affect the conserved residues Arg1075, Leu2963 and Ile3253, respectively.** Protein alignment demonstrates that Arg1075 is conserved throughout the listed species. Leu2963 is replaced by a chemically similar residue in chicken and Ile3253 is replaced by the chemically similar valine in drosophila and leucine in the nematode.
(TIF)

Table S1 **Homozygosity mapping for AHE6.**
(DOCX)

Table S2 **Primer sequences and PCR conditions.**
(DOCX)

Table S3 **Coverage of the congenital myopathy genes for families 1 and 2.**
(DOCX)

Table S4 **Coverage of the congenital myopathy genes for families 3-6.**
(DOCX)

Table S5 **Web resources.**
(DOCX)
