# Supplementary figures and images for: An Integrated Diagnosis Strategy for Congenital Myopathies
Source: PLoS One. 2013 Jun 24;8(6):e67527. doi: 10.1371/journal.pone.0067527 (PMC3691193; doi:10.1371/journal.pone.0067527)

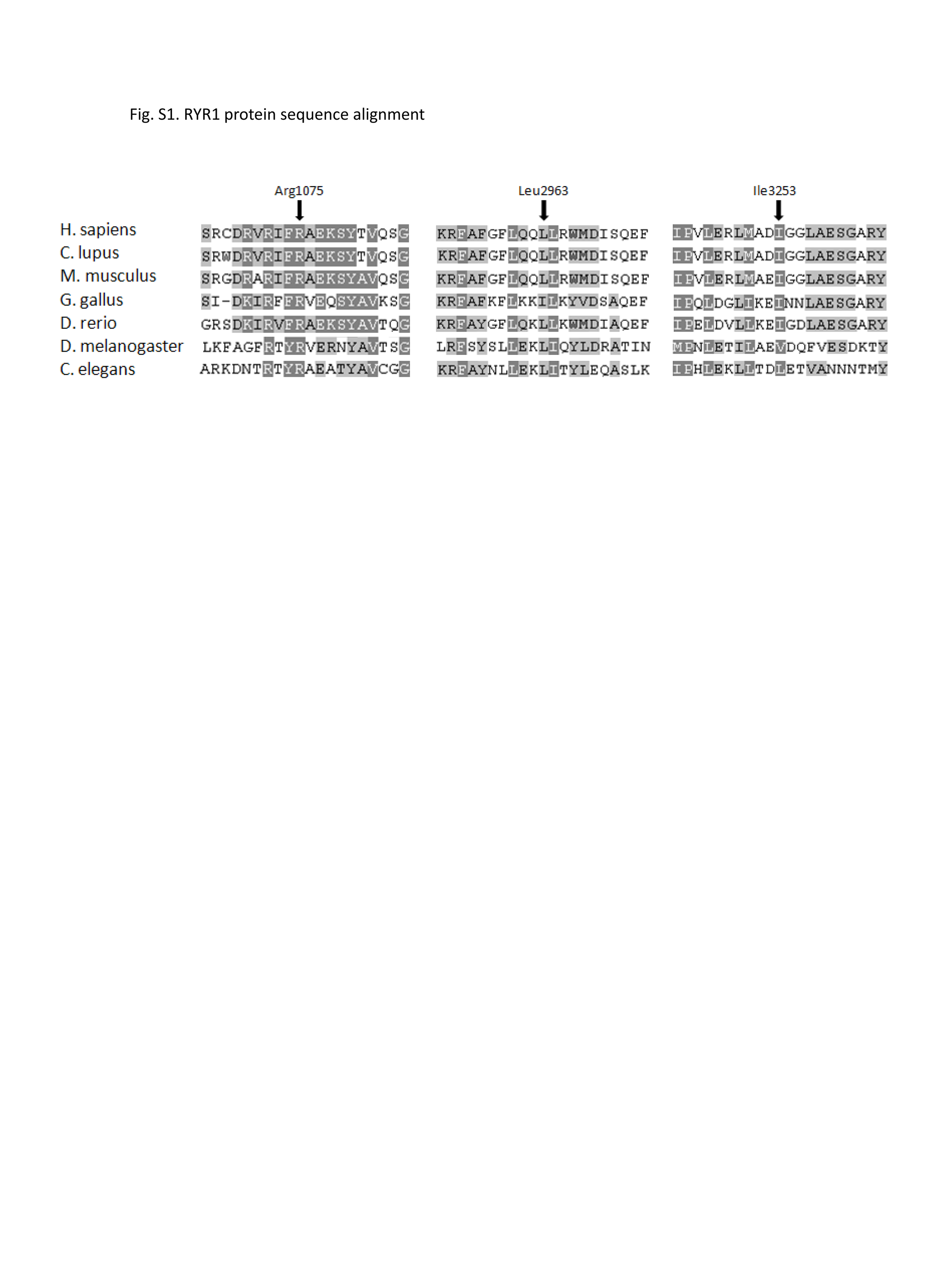

Supplement: Figure S1 — Gomori trichrome staining of a tibialis anterior muscle section from patient ARX33 revealed the presence of nemaline rods. (TIF) [file pone.0067527.s001.tif]

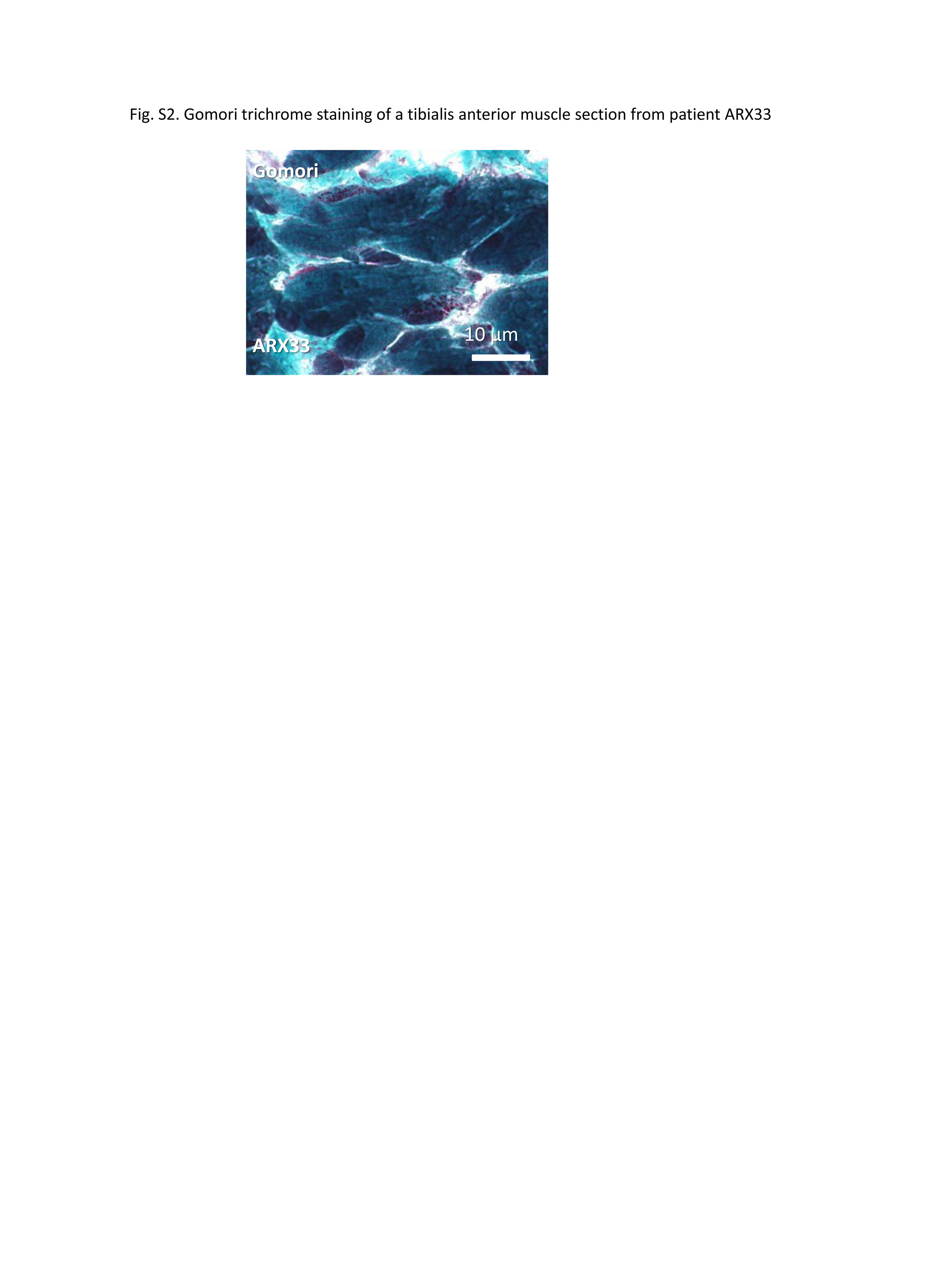

Supplement: Figure S2 — The novel RYR1 mutations c.3223C>T (Family 3), c.8888T>C (Family 6) and c.9758T>C (Family 5) affect the conserved residues Arg1075, Leu2963 and Ile3253, respectively. Protein alignment demonstrates that Arg1075 is conserved throughout the listed species. Leu2963 is replaced by a chemically similar residue in chicken and Ile3253 is replaced by the chemically similar valine in drosophila and leucine in the nematode. (TIF) [file pone.0067527.s002.tif]
