# Supplementary material for: An Integrated Diagnosis Strategy for Congenital Myopathies
Source: PLoS One. 2013 Jun 24;8(6):e67527. doi: 10.1371/journal.pone.0067527 (PMC3691193; doi:10.1371/journal.pone.0067527)
Supplement: Table S1 — Homozygosity mapping for AHE6. (DOCX) [file pone.0067527.s003.docx]

**Table S1:** Homozygosity mapping for AHE6

| Chromosome | Homozygous regions  (Positions in Mb) | Number of genes |
| --- | --- | --- |
| 1 | 33.9 - 43.5 | 238 |
|  | 60.4 - 86.4 | 325 |
|  | 95.9 - 120.1 | 423 |
|  | 193.6 - 196.0 | 12 |
|  | 236.6 - 240.5 | 54 |
| 2 | 97.0 - 99.7 | 59 |
|  | 207.8 - 227.4 | 316 |
|  | 239.3 - 244.0 | 87 |
| 3 | 45.9 - 73.4 | 479 |
| 4 | 0.7 - 30.6 | 439 |
|  | 182.5 - 190.0 | 128 |
| 5 | 10.3 - 33.7 | 259 |
|  | 54.0 - 67.4 | 186 |
|  | 163.0 - 169.8 | 63 |
| 6 | 38.8 - 58.0 | 339 |
|  | 62.0 - 71.0 | 56 |
|  | 153.8 - 166.0 | 146 |
| 7 | 7.5 - 25.4 | 210 |
|  | 61.0 - 67.0 | 190 |
|  | 105.8 - 111.9 | 63 |
| 9 | 129.8 - 135.0 | 171 |
| 11 | 74.0 - 174.0 | 526 |
|  | 126.8 - 132.2 | 74 |
| 12 | 4.2 - 18.1 | 434 |
|  | 39.7 - 44.2 | 66 |
| 13 | 40.8 - 44.7 | 64 |
|  | 54.0 - 64.1 | 49 |
|  | 80.2 - 91.0 | 57 |
| 14 | 42.3 - 89.7 | 790 |
|  | 98.2 - 107.0 | 450 |
| 16 | 48.5 - 53.5 | 556 |
|  | 72.5 - 77.5 | 57 |
| 17 | 3.6 - 12.6 | 247 |
|  | 68.0 - 73.0 | 64 |
| 19 | 3.0 - 24.0 | 664 |
|  | 32.5 - 53.6 | 803 |
| 20 | 1.9 - 15.5 | 212 |
