# Supplementary material for: An Integrated Diagnosis Strategy for Congenital Myopathies
Source: PLoS One. 2013 Jun 24;8(6):e67527. doi: 10.1371/journal.pone.0067527 (PMC3691193; doi:10.1371/journal.pone.0067527)
Supplement: Table S2 — Primer sequences and PCR conditions. (DOCX) [file pone.0067527.s004.docx]

**Table S2:** Primer sequences and PCR conditions

| Amplicon | Forward Primer (5’-3’) | Reverse Primer (5’-3’) | Size (nt) | MgCl_2_ | Ann. T° |
| --- | --- | --- | --- | --- | --- |
| NEB x45 | CAAAGCTCACATCACCTAGC | GAGTGACCATAAGCAATGACG | 464 | 2 mM | 58°C |
| NEB x46 | CACACACTTAGGATTCTTGGG | GGGCATCACACTTGAGGTTA | 385 | 2 mM | 58°C |
| NEB x58 | GACAAATTTGTCACTTGCAC | CCTTTGCTTGGTACTAATGT | 343 | 2 mM | 58°C |
| NEB x122 | AGGCACATGTAAGTTGGCAC | GGGATGGACACTCTTTCCCT | 486 | 2 mM | 58°C |
| NEBx44-47  (cDNA) | AAGGCTGCAAGAGCCTCTAG | GTCCACATCATGCCTGATATC | 615 | 2 mM | 58°C |
| NEBx57-60  (cDNA) | AAGAACTACCTGCACCAGTG | GACCAAGTTCAGCAGCCCA | 666 | 2 mM | 58°C |
| NEBx120-124  (cDNA) | CAGTACATCCTCAGTGACC | CCGAGTTGGCAAGCTTAAC | 641 | 2 mM | 58°C |
| RYR1x4 | CGGGGATCTGTGCTTATTCT | TAGAAGGAGGCTGACCTCCC | 188 | 2 mM | 58°C |
| RYR1x25 | CCAACTTCTCGATGTCTTGG | CACAGCTTGTCTACTCTGG | 338 | 2 mM | 58°C |
| RYR1x43 | CAGAGCTGAACCGGACTGA | GTCCAGGCAGGAATCCCT | 364 | 2 mM | 64°C |
| RYR1x48 | GGATCCTTTGGCCACAGTC | GGGCAGGCTTCAGGGTG | 334 | 2 mM | 58°C |
| RYR1x51 | GGTCAGTAAGGCTTATAGCG | CTCAAGACCCCAGCTTTCC | 438 | 2 mM | 58°C |
| RYR1x58 | GGGTGAAGCCAAAGCTGATA | CTGTTCATGCACTCAATCCG | 329 | 2 mM | 58°C |
| RYR1x59 | AGCATCCTTCCCATCAGAAC | CATGGAGGCTCTGTCCTG | 437 | 2 mM | 58°C |
| RYR1x66 | TGGTGTCTGATGTATTGCG | CTTGGCAGATGGTCTGAAAG | 493 | 1.5 mM | 56°C |
