# Supplementary material for: An Integrated Diagnosis Strategy for Congenital Myopathies
Source: PLoS One. 2013 Jun 24;8(6):e67527. doi: 10.1371/journal.pone.0067527 (PMC3691193; doi:10.1371/journal.pone.0067527)
Supplement: Table S3 — Coverage of the congenital myopathy genes for families 1 and 2. (DOCX) [file pone.0067527.s005.docx]

**Supplementary Table 3:** Coverage of the congenital myopathy genes for families 1 and 2

| **Chr** | **Start** | **End** | **Gene** | **GC (%)** | **Read number ARX30** | **Read number ARX33** |
| --- | --- | --- | --- | --- | --- | --- |
| chr1 | 229567167 | 229567678 | ACTA1 | 0.63 | 225 | 246 |
| chr1 | 229567722 | 229567956 | ACTA1 | 0.65 | 62 | 44 |
| chr1 | 229568014 | 229568217 | ACTA1 | 0.66 | 34 | 34 |
| chr1 | 229568259 | 229568644 | ACTA1 | 0.63 | 273 | 305 |
| chr1 | 229568677 | 229568917 | ACTA1 | 0.68 | 90 | 124 |
| chr2 | 127806065 | 127806215 | BIN1 | 0.64 | 83 | 120 |
| chr2 | 127807987 | 127808107 | BIN1 | 0.61 | 48 | 80 |
| chr2 | 127808342 | 127808492 | BIN1 | 0.64 | 76 | 70 |
| chr2 | 127808684 | 127808834 | BIN1 | 0.66 | 28 | 33 |
| chr2 | 127809824 | 127809944 | BIN1 | 0.70 | 3 | 5 |
| chr2 | 127810919 | 127811069 | BIN1 | 0.58 | 34 | 35 |
| chr2 | 127811474 | 127811594 | BIN1 | 0.66 | 1 | 5 |
| chr2 | 127814992 | 127815232 | BIN1 | 0.68 | 7 | 2 |
| chr2 | 127816489 | 127816609 | BIN1 | 0.60 | 6 | 7 |
| chr2 | 127816638 | 127816758 | BIN1 | 0.63 | 8 | 8 |
| chr2 | 127818103 | 127818253 | BIN1 | 0.62 | 37 | 27 |
| chr2 | 127819671 | 127819791 | BIN1 | 0.61 | 11 | 15 |
| chr2 | 127821094 | 127821244 | BIN1 | 0.62 | 38 | 31 |
| chr2 | 127821461 | 127821611 | BIN1 | 0.64 | 49 | 57 |
| chr2 | 127825724 | 127825844 | BIN1 | 0.59 | 29 | 27 |
| chr2 | 127826463 | 127826613 | BIN1 | 0.61 | 60 | 52 |
| chr2 | 127827558 | 127827678 | BIN1 | 0.59 | 42 | 45 |
| chr2 | 127828115 | 127828235 | BIN1 | 0.58 | 138 | 165 |
| chr2 | 127828274 | 127828424 | BIN1 | 0.65 | 67 | 78 |
| chr2 | 127834151 | 127834301 | BIN1 | 0.54 | 257 | 252 |
| chr2 | 127864417 | 127864537 | BIN1 | 0.68 | 20 | 33 |
| chr14 | 35182066 | 35182186 | CFL2 | 0.37 | 209 | 186 |
| chr14 | 35182243 | 35182363 | CFL2 | 0.32 | 90 | 83 |
| chr14 | 35182433 | 35182793 | CFL2 | 0.36 | 399 | 398 |
| chr14 | 35182820 | 35182940 | CFL2 | 0.39 | 125 | 149 |
| chr14 | 35183684 | 35183804 | CFL2 | 0.74 | 9 | 10 |
| chr12 | 41302204 | 41302324 | CNTN1 | 0.32 | 176 | 156 |
| chr12 | 41303830 | 41303950 | CNTN1 | 0.31 | 278 | 230 |
| chr12 | 41312386 | 41312626 | CNTN1 | 0.39 | 249 | 245 |
| chr12 | 41316023 | 41316263 | CNTN1 | 0.39 | 214 | 215 |
| chr12 | 41318346 | 41318496 | CNTN1 | 0.42 | 113 | 141 |
| chr12 | 41323580 | 41323820 | CNTN1 | 0.38 | 286 | 324 |
| chr12 | 41327252 | 41327372 | CNTN1 | 0.35 | 345 | 304 |
| chr12 | 41327439 | 41327709 | CNTN1 | 0.36 | 455 | 365 |
| chr12 | 41330524 | 41330764 | CNTN1 | 0.44 | 323 | 261 |
| chr12 | 41331310 | 41331550 | CNTN1 | 0.31 | 222 | 199 |
| chr12 | 41333091 | 41333331 | CNTN1 | 0.38 | 181 | 176 |
| chr12 | 41337342 | 41337582 | CNTN1 | 0.31 | 315 | 266 |
| chr12 | 41337764 | 41338004 | CNTN1 | 0.37 | 256 | 239 |
| chr12 | 41352855 | 41353095 | CNTN1 | 0.37 | 162 | 160 |
| chr12 | 41365215 | 41365335 | CNTN1 | 0.48 | 110 | 137 |
| chr12 | 41374639 | 41374909 | CNTN1 | 0.40 | 531 | 626 |
| chr12 | 41386876 | 41387116 | CNTN1 | 0.36 | 340 | 328 |
| chr12 | 41408004 | 41408124 | CNTN1 | 0.43 | 109 | 115 |
| chr12 | 41410480 | 41410720 | CNTN1 | 0.40 | 278 | 228 |
| chr12 | 41414100 | 41414250 | CNTN1 | 0.34 | 228 | 210 |
| chr12 | 41418894 | 41419164 | CNTN1 | 0.46 | 751 | 654 |
| chr12 | 41421654 | 41421774 | CNTN1 | 0.45 | 95 | 118 |
| chr12 | 41422822 | 41423062 | CNTN1 | 0.41 | 409 | 354 |
| chr12 | 41463738 | 41463858 | CNTN1 | 0.50 | 56 | 97 |
| chr19 | 10828999 | 10829233 | DNM2 | 0.77 | 7 | 17 |
| chr19 | 10870360 | 10870510 | DNM2 | 0.58 | 233 | 209 |
| chr19 | 10883079 | 10883349 | DNM2 | 0.54 | 218 | 235 |
| chr19 | 10886330 | 10886600 | DNM2 | 0.59 | 385 | 387 |
| chr19 | 10887752 | 10887902 | DNM2 | 0.59 | 67 | 60 |
| chr19 | 10893565 | 10893835 | DNM2 | 0.60 | 125 | 124 |
| chr19 | 10897190 | 10897430 | DNM2 | 0.61 | 137 | 152 |
| chr19 | 10904313 | 10904583 | DNM2 | 0.56 | 310 | 367 |
| chr19 | 10906021 | 10906141 | DNM2 | 0.50 | 108 | 99 |
| chr19 | 10906655 | 10906925 | DNM2 | 0.54 | 369 | 418 |
| chr19 | 10907974 | 10908244 | DNM2 | 0.54 | 154 | 128 |
| chr19 | 10909141 | 10909261 | DNM2 | 0.59 | 62 | 60 |
| chr19 | 10912908 | 10913058 | DNM2 | 0.54 | 123 | 85 |
| chr19 | 10916557 | 10916677 | DNM2 | 0.57 | 79 | 86 |
| chr19 | 10919190 | 10919329 | DNM2 | 0.47 | 89 | 78 |
| chr19 | 10922374 | 10922494 | DNM2 | 0.58 | 54 | 65 |
| chr19 | 10922936 | 10923056 | DNM2 | 0.57 | 9 | 13 |
| chr19 | 10930620 | 10930770 | DNM2 | 0.54 | 183 | 171 |
| chr19 | 10934429 | 10934579 | DNM2 | 0.64 | 71 | 61 |
| chr19 | 10935664 | 10935927 | DNM2 | 0.56 | 220 | 254 |
| chr19 | 10939677 | 10939947 | DNM2 | 0.63 | 111 | 106 |
| chr19 | 10940718 | 10941108 | DNM2 | 0.67 | 245 | 272 |
| chr19 | 10941628 | 10941748 | DNM2 | 0.66 | 5 | 9 |
| chrX | 149761047 | 149761167 | MTM1 | 0.36 | 208 | 87 |
| chrX | 149764937 | 149765057 | MTM1 | 0.45 | 99 | 53 |
| chrX | 149767042 | 149767162 | MTM1 | 0.30 | 150 | 75 |
| chrX | 149783056 | 149783176 | MTM1 | 0.38 | 101 | 61 |
| chrX | 149787471 | 149787621 | MTM1 | 0.48 | 145 | 76 |
| chrX | 149807397 | 149807547 | MTM1 | 0.36 | 362 | 197 |
| chrX | 149809666 | 149809936 | MTM1 | 0.44 | 277 | 159 |
| chrX | 149814129 | 149814369 | MTM1 | 0.41 | 338 | 173 |
| chrX | 149818131 | 149818401 | MTM1 | 0.32 | 517 | 250 |
| chrX | 149826276 | 149826516 | MTM1 | 0.43 | 251 | 129 |
| chrX | 149828092 | 149828242 | MTM1 | 0.34 | 131 | 68 |
| chrX | 149828810 | 149828960 | MTM1 | 0.33 | 155 | 79 |
| chrX | 149831873 | 149832113 | MTM1 | 0.38 | 346 | 188 |
| chrX | 149839834 | 149840104 | MTM1 | 0.52 | 201 | 99 |
| chr3 | 9691226 | 9691466 | MTMR14 | 0.71 | 25 | 19 |
| chr3 | 9695258 | 9695498 | MTMR14 | 0.51 | 245 | 232 |
| chr3 | 9703944 | 9704064 | MTMR14 | 0.54 | 71 | 77 |
| chr3 | 9710380 | 9710500 | MTMR14 | 0.49 | 134 | 119 |
| chr3 | 9711055 | 9711205 | MTMR14 | 0.62 | 65 | 57 |
| chr3 | 9712672 | 9712906 | MTMR14 | 0.46 | 167 | 167 |
| chr3 | 9714345 | 9714465 | MTMR14 | 0.53 | 135 | 134 |
| chr3 | 9717261 | 9717381 | MTMR14 | 0.45 | 313 | 304 |
| chr3 | 9718945 | 9719095 | MTMR14 | 0.42 | 179 | 171 |
| chr3 | 9719644 | 9719764 | MTMR14 | 0.54 | 83 | 96 |
| chr3 | 9724804 | 9724954 | MTMR14 | 0.41 | 111 | 98 |
| chr3 | 9726221 | 9726371 | MTMR14 | 0.59 | 134 | 120 |
| chr3 | 9726519 | 9726669 | MTMR14 | 0.56 | 76 | 103 |
| chr3 | 9726822 | 9726972 | MTMR14 | 0.54 | 123 | 142 |
| chr3 | 9729479 | 9729599 | MTMR14 | 0.47 | 13 | 17 |
| chr3 | 9730348 | 9730468 | MTMR14 | 0.55 | 84 | 71 |
| chr3 | 9730576 | 9730816 | MTMR14 | 0.61 | 136 | 161 |
| chr3 | 9731617 | 9731857 | MTMR14 | 0.59 | 61 | 87 |
| chr3 | 9739352 | 9739592 | MTMR14 | 0.58 | 407 | 437 |
| chr3 | 9743415 | 9743685 | MTMR14 | 0.59 | 200 | 201 |
| chr14 | 23881981 | 23882131 | MYH7 | 0.52 | 62 | 79 |
| chr14 | 23882884 | 23883154 | MYH7 | 0.62 | 148 | 140 |
| chr14 | 23883173 | 23883323 | MYH7 | 0.64 | 108 | 117 |
| chr14 | 23884161 | 23884521 | MYH7 | 0.62 | 243 | 349 |
| chr14 | 23884532 | 23884772 | MYH7 | 0.57 | 176 | 269 |
| chr14 | 23884789 | 23885059 | MYH7 | 0.62 | 162 | 186 |
| chr14 | 23885186 | 23885546 | MYH7 | 0.62 | 322 | 334 |
| chr14 | 23886018 | 23886258 | MYH7 | 0.60 | 200 | 221 |
| chr14 | 23886294 | 23886564 | MYH7 | 0.61 | 229 | 308 |
| chr14 | 23886683 | 23886923 | MYH7 | 0.63 | 129 | 156 |
| chr14 | 23887366 | 23887636 | MYH7 | 0.66 | 158 | 180 |
| chr14 | 23888294 | 23888564 | MYH7 | 0.59 | 209 | 163 |
| chr14 | 23888604 | 23888874 | MYH7 | 0.59 | 281 | 318 |
| chr14 | 23888986 | 23889408 | MYH7 | 0.64 | 133 | 133 |
| chr14 | 23890151 | 23890271 | MYH7 | 0.55 | 68 | 70 |
| chr14 | 23891341 | 23891581 | MYH7 | 0.59 | 107 | 103 |
| chr14 | 23892723 | 23892963 | MYH7 | 0.53 | 298 | 332 |
| chr14 | 23893056 | 23893416 | MYH7 | 0.53 | 225 | 235 |
| chr14 | 23893895 | 23894285 | MYH7 | 0.58 | 166 | 231 |
| chr14 | 23894438 | 23894678 | MYH7 | 0.59 | 154 | 179 |
| chr14 | 23894845 | 23895320 | MYH7 | 0.55 | 288 | 319 |
| chr14 | 23895969 | 23896089 | MYH7 | 0.48 | 53 | 54 |
| chr14 | 23896392 | 23896542 | MYH7 | 0.57 | 60 | 65 |
| chr14 | 23896738 | 23897128 | MYH7 | 0.55 | 203 | 222 |
| chr14 | 23897673 | 23897913 | MYH7 | 0.54 | 177 | 206 |
| chr14 | 23898118 | 23898358 | MYH7 | 0.55 | 187 | 221 |
| chr14 | 23898376 | 23898601 | MYH7 | 0.56 | 241 | 238 |
| chr14 | 23898932 | 23899172 | MYH7 | 0.54 | 169 | 200 |
| chr14 | 23899760 | 23899880 | MYH7 | 0.54 | 45 | 42 |
| chr14 | 23900068 | 23900218 | MYH7 | 0.44 | 230 | 197 |
| chr14 | 23900568 | 23900718 | MYH7 | 0.49 | 270 | 303 |
| chr14 | 23900749 | 23900899 | MYH7 | 0.62 | 251 | 264 |
| chr14 | 23900963 | 23901083 | MYH7 | 0.58 | 118 | 134 |
| chr14 | 23901641 | 23901761 | MYH7 | 0.54 | 70 | 94 |
| chr14 | 23901805 | 23902045 | MYH7 | 0.59 | 155 | 211 |
| chr14 | 23902244 | 23902484 | MYH7 | 0.59 | 135 | 117 |
| chr14 | 23902720 | 23902960 | MYH7 | 0.57 | 150 | 163 |
| chr2 | 152342240 | 152342480 | NEB | 0.46 | 717 | 696 |
| chr2 | 152346417 | 152346657 | NEB | 0.35 | 796 | 880 |
| chr2 | 152346838 | 152347078 | NEB | 0.56 | 112 | 139 |
| chr2 | 152348182 | 152348302 | NEB | 0.44 | 88 | 129 |
| chr2 | 152348583 | 152348823 | NEB | 0.42 | 651 | 665 |
| chr2 | 152348834 | 152349074 | NEB | 0.47 | 441 | 432 |
| chr2 | 152349849 | 152349972 | NEB | 0.43 | 187 | 183 |
| chr2 | 152350274 | 152350394 | NEB | 0.42 | 133 | 129 |
| chr2 | 152350660 | 152350780 | NEB | 0.45 | 184 | 186 |
| chr2 | 152352775 | 152352895 | NEB | 0.45 | 372 | 376 |
| chr2 | 152353055 | 152353175 | NEB | 0.47 | 88 | 98 |
| chr2 | 152353440 | 152353560 | NEB | 0.44 | 417 | 383 |
| chr2 | 152354125 | 152354245 | NEB | 0.42 | 514 | 434 |
| chr2 | 152354759 | 152354879 | NEB | 0.42 | 163 | 169 |
| chr2 | 152355810 | 152355930 | NEB | 0.40 | 154 | 182 |
| chr2 | 152357890 | 152358010 | NEB | 0.45 | 237 | 196 |
| chr2 | 152359292 | 152359412 | NEB | 0.42 | 304 | 282 |
| chr2 | 152359848 | 152359968 | NEB | 0.37 | 223 | 227 |
| chr2 | 152361977 | 152362097 | NEB | 0.47 | 380 | 379 |
| chr2 | 152362665 | 152362785 | NEB | 0.45 | 420 | 401 |
| chr2 | 152363354 | 152363594 | NEB | 0.31 | 509 | 456 |
| chr2 | 152364450 | 152364690 | NEB | 0.45 | 225 | 244 |
| chr2 | 152369178 | 152369418 | NEB | 0.39 | 738 | 631 |
| chr2 | 152370030 | 152370270 | NEB | 0.39 | 319 | 291 |
| chr2 | 152370766 | 152371006 | NEB | 0.43 | 720 | 762 |
| chr2 | 152371266 | 152371506 | NEB | 0.36 | 240 | 226 |
| chr2 | 152372904 | 152373144 | NEB | 0.43 | 424 | 386 |
| chr2 | 152374763 | 152375003 | NEB | 0.43 | 700 | 684 |
| chr2 | 152375410 | 152375650 | NEB | 0.41 | 368 | 355 |
| chr2 | 152376158 | 152376346 | NEB | 0.35 | 697 | 647 |
| chr2 | 152380756 | 152381167 | NEB | 0.51 | 696 | 727 |
| chr2 | 152381611 | 152381851 | NEB | 0.43 | 498 | 530 |
| chr2 | 152382406 | 152382843 | NEB | 0.50 | 626 | 694 |
| chr2 | 152383363 | 152383603 | NEB | 0.47 | 274 | 268 |
| chr2 | 152383926 | 152384166 | NEB | 0.44 | 299 | 314 |
| chr2 | 152385655 | 152385895 | NEB | 0.41 | 340 | 336 |
| chr2 | 152387441 | 152387681 | NEB | 0.39 | 498 | 467 |
| chr2 | 152388269 | 152388477 | NEB | 0.36 | 372 | 329 |
| chr2 | 152389887 | 152390127 | NEB | 0.41 | 182 | 253 |
| chr2 | 152390660 | 152390900 | NEB | 0.49 | 475 | 532 |
| chr2 | 152392136 | 152392376 | NEB | 0.34 | 348 | 369 |
| chr2 | 152393577 | 152393817 | NEB | 0.39 | 508 | 562 |
| chr2 | 152394317 | 152394557 | NEB | 0.38 | 402 | 335 |
| chr2 | 152394585 | 152394825 | NEB | 0.34 | 480 | 502 |
| chr2 | 152396789 | 152397029 | NEB | 0.42 | 223 | 195 |
| chr2 | 152397142 | 152397382 | NEB | 0.42 | 362 | 394 |
| chr2 | 152397892 | 152398117 | NEB | 0.47 | 449 | 415 |
| chr2 | 152402336 | 152402576 | NEB | 0.34 | 350 | 326 |
| chr2 | 152402847 | 152402967 | NEB | 0.54 | 256 | 241 |
| chr2 | 152403874 | 152404319 | NEB | 0.44 | 775 | 895 |
| chr2 | 152404755 | 152404995 | NEB | 0.42 | 448 | 498 |
| chr2 | 152406081 | 152406321 | NEB | 0.46 | 129 | 143 |
| chr2 | 152408185 | 152408425 | NEB | 0.49 | 203 | 226 |
| chr2 | 152409117 | 152409357 | NEB | 0.45 | 233 | 276 |
| chr2 | 152409843 | 152410083 | NEB | 0.37 | 372 | 371 |
| chr2 | 152410320 | 152410560 | NEB | 0.45 | 563 | 557 |
| chr2 | 152411437 | 152411622 | NEB | 0.36 | 330 | 309 |
| chr2 | 152417039 | 152417279 | NEB | 0.51 | 204 | 154 |
| chr2 | 152417449 | 152417893 | NEB | 0.39 | 557 | 606 |
| chr2 | 152418555 | 152418795 | NEB | 0.43 | 684 | 619 |
| chr2 | 152419103 | 152419343 | NEB | 0.43 | 215 | 250 |
| chr2 | 152420056 | 152420510 | NEB | 0.45 | 1002 | 1036 |
| chr2 | 152421489 | 152421682 | NEB | 0.33 | 352 | 328 |
| chr2 | 152421946 | 152422401 | NEB | 0.44 | 796 | 804 |
| chr2 | 152423657 | 152424017 | NEB | 0.42 | 728 | 709 |
| chr2 | 152424518 | 152424758 | NEB | 0.47 | 345 | 368 |
| chr2 | 152424760 | 152425000 | NEB | 0.46 | 386 | 411 |
| chr2 | 152425120 | 152425240 | NEB | 0.39 | 340 | 305 |
| chr2 | 152425713 | 152425953 | NEB | 0.45 | 470 | 445 |
| chr2 | 152426570 | 152426930 | NEB | 0.52 | 256 | 275 |
| chr2 | 152426942 | 152427182 | NEB | 0.48 | 193 | 219 |
| chr2 | 152432140 | 152432380 | NEB | 0.37 | 781 | 820 |
| chr2 | 152432646 | 152432886 | NEB | 0.44 | 355 | 324 |
| chr2 | 152435827 | 152436187 | NEB | 0.50 | 95 | 172 |
| chr2 | 152437244 | 152437484 | NEB | 0.47 | 0 | 0 |
| chr2 | 152437928 | 152438168 | NEB | 0.41 | 30 | 22 |
| chr2 | 152438959 | 152439199 | NEB | 0.46 | 0 | 0 |
| chr2 | 152439930 | 152440170 | NEB | 0.46 | 0 | 0 |
| chr2 | 152446378 | 152446738 | NEB | 0.49 | 0 | 0 |
| chr2 | 152447795 | 152448035 | NEB | 0.47 | 237 | 278 |
| chr2 | 152448479 | 152448719 | NEB | 0.41 | 335 | 355 |
| chr2 | 152449511 | 152449751 | NEB | 0.46 | 0 | 0 |
| chr2 | 152450482 | 152450722 | NEB | 0.46 | 3 | 3 |
| chr2 | 152456931 | 152457291 | NEB | 0.49 | 0 | 0 |
| chr2 | 152458348 | 152458588 | NEB | 0.47 | 0 | 0 |
| chr2 | 152459032 | 152459272 | NEB | 0.41 | 0 | 0 |
| chr2 | 152460064 | 152460304 | NEB | 0.47 | 182 | 324 |
| chr2 | 152461035 | 152461275 | NEB | 0.46 | 0 | 0 |
| chr2 | 152466298 | 152466658 | NEB | 0.42 | 741 | 799 |
| chr2 | 152466963 | 152467203 | NEB | 0.47 | 582 | 530 |
| chr2 | 152467207 | 152467447 | NEB | 0.45 | 528 | 526 |
| chr2 | 152468681 | 152468921 | NEB | 0.46 | 441 | 445 |
| chr2 | 152470765 | 152471125 | NEB | 0.49 | 816 | 884 |
| chr2 | 152472449 | 152472689 | NEB | 0.37 | 502 | 428 |
| chr2 | 152473809 | 152474049 | NEB | 0.34 | 339 | 308 |
| chr2 | 152474770 | 152475010 | NEB | 0.43 | 914 | 856 |
| chr2 | 152475940 | 152476300 | NEB | 0.46 | 619 | 706 |
| chr2 | 152477386 | 152477606 | NEB | 0.44 | 290 | 288 |
| chr2 | 152481979 | 152482219 | NEB | 0.35 | 438 | 399 |
| chr2 | 152483497 | 152483737 | NEB | 0.48 | 582 | 574 |
| chr2 | 152484012 | 152484372 | NEB | 0.50 | 1149 | 1417 |
| chr2 | 152485986 | 152486226 | NEB | 0.41 | 382 | 435 |
| chr2 | 152487160 | 152487400 | NEB | 0.36 | 623 | 631 |
| chr2 | 152487614 | 152487734 | NEB | 0.45 | 505 | 600 |
| chr2 | 152490264 | 152490383 | NEB | 0.53 | 565 | 609 |
| chr2 | 152496352 | 152496610 | NEB | 0.40 | 691 | 583 |
| chr2 | 152496844 | 152497204 | NEB | 0.52 | 1017 | 1062 |
| chr2 | 152499021 | 152499450 | NEB | 0.37 | 687 | 807 |
| chr2 | 152499645 | 152499885 | NEB | 0.40 | 603 | 568 |
| chr2 | 152500307 | 152500667 | NEB | 0.52 | 1192 | 1307 |
| chr2 | 152500915 | 152501155 | NEB | 0.37 | 580 | 576 |
| chr2 | 152502575 | 152502815 | NEB | 0.33 | 412 | 341 |
| chr2 | 152506671 | 152506911 | NEB | 0.44 | 619 | 564 |
| chr2 | 152507063 | 152507423 | NEB | 0.43 | 1544 | 1443 |
| chr2 | 152510439 | 152510679 | NEB | 0.34 | 514 | 452 |
| chr2 | 152511775 | 152511902 | NEB | 0.35 | 299 | 288 |
| chr2 | 152512313 | 152512553 | NEB | 0.45 | 466 | 433 |
| chr2 | 152512642 | 152513002 | NEB | 0.43 | 1284 | 1245 |
| chr2 | 152514430 | 152514670 | NEB | 0.41 | 366 | 365 |
| chr2 | 152515510 | 152515750 | NEB | 0.36 | 868 | 884 |
| chr2 | 152518631 | 152518871 | NEB | 0.43 | 618 | 534 |
| chr2 | 152520037 | 152520397 | NEB | 0.48 | 485 | 523 |
| chr2 | 152520948 | 152521188 | NEB | 0.43 | 382 | 442 |
| chr2 | 152521204 | 152521444 | NEB | 0.43 | 417 | 499 |
| chr2 | 152521829 | 152522069 | NEB | 0.46 | 325 | 342 |
| chr2 | 152522579 | 152522939 | NEB | 0.46 | 845 | 918 |
| chr2 | 152524273 | 152524491 | NEB | 0.34 | 464 | 453 |
| chr2 | 152525472 | 152525712 | NEB | 0.41 | 176 | 192 |
| chr2 | 152527519 | 152527759 | NEB | 0.46 | 412 | 420 |
| chr2 | 152528858 | 152529218 | NEB | 0.45 | 825 | 734 |
| chr2 | 152530924 | 152531164 | NEB | 0.41 | 411 | 414 |
| chr2 | 152531758 | 152531972 | NEB | 0.37 | 488 | 478 |
| chr2 | 152534061 | 152534301 | NEB | 0.44 | 804 | 848 |
| chr2 | 152534365 | 152534725 | NEB | 0.41 | 951 | 1003 |
| chr2 | 152536168 | 152536603 | NEB | 0.44 | 571 | 603 |
| chr2 | 152537232 | 152537352 | NEB | 0.40 | 534 | 570 |
| chr2 | 152539109 | 152539349 | NEB | 0.43 | 423 | 465 |
| chr2 | 152541270 | 152541510 | NEB | 0.43 | 405 | 388 |
| chr2 | 152543869 | 152544313 | NEB | 0.45 | 660 | 721 |
| chr2 | 152544737 | 152544977 | NEB | 0.36 | 358 | 376 |
| chr2 | 152547229 | 152547349 | NEB | 0.43 | 44 | 68 |
| chr2 | 152548309 | 152548945 | NEB | 0.35 | 1421 | 1329 |
| chr2 | 152550773 | 152551209 | NEB | 0.44 | 827 | 805 |
| chr2 | 152552023 | 152552263 | NEB | 0.37 | 462 | 473 |
| chr2 | 152553139 | 152553259 | NEB | 0.45 | 170 | 202 |
| chr2 | 152553593 | 152554229 | NEB | 0.36 | 1489 | 1444 |
| chr2 | 152563332 | 152563572 | NEB | 0.37 | 340 | 389 |
| chr2 | 152566103 | 152566343 | NEB | 0.38 | 222 | 221 |
| chr2 | 152566879 | 152567119 | NEB | 0.37 | 309 | 334 |
| chr2 | 152573861 | 152574101 | NEB | 0.39 | 456 | 455 |
| chr2 | 152579827 | 152580067 | NEB | 0.43 | 320 | 265 |
| chr2 | 152580705 | 152580945 | NEB | 0.46 | 227 | 190 |
| chr2 | 152581302 | 152581542 | NEB | 0.40 | 771 | 808 |
| chr2 | 152581900 | 152582140 | NEB | 0.41 | 480 | 430 |
| chr2 | 152584192 | 152584432 | NEB | 0.45 | 437 | 499 |
| chr2 | 152586089 | 152586209 | NEB | 0.47 | 247 | 248 |
| chr2 | 152589592 | 152589712 | NEB | 0.37 | 269 | 236 |
| chr19 | 38924431 | 38924551 | RYR1 | 0.62 | 94 | 89 |
| chr19 | 38931324 | 38931564 | RYR1 | 0.66 | 21 | 40 |
| chr19 | 38932950 | 38933100 | RYR1 | 0.62 | 94 | 111 |
| chr19 | 38934174 | 38934294 | RYR1 | 0.66 | 38 | 64 |
| chr19 | 38934306 | 38934456 | RYR1 | 0.61 | 88 | 116 |
| chr19 | 38934754 | 38934904 | RYR1 | 0.59 | 74 | 73 |
| chr19 | 38935180 | 38935330 | RYR1 | 0.63 | 45 | 34 |
| chr19 | 38937098 | 38937218 | RYR1 | 0.58 | 79 | 69 |
| chr19 | 38937280 | 38937430 | RYR1 | 0.60 | 120 | 97 |
| chr19 | 38938922 | 38939192 | RYR1 | 0.63 | 215 | 224 |
| chr19 | 38939220 | 38939457 | RYR1 | 0.64 | 111 | 122 |
| chr19 | 38942314 | 38942584 | RYR1 | 0.63 | 140 | 156 |
| chr19 | 38943436 | 38943676 | RYR1 | 0.66 | 32 | 30 |
| chr19 | 38945822 | 38946198 | RYR1 | 0.55 | 302 | 339 |
| chr19 | 38946211 | 38946451 | RYR1 | 0.58 | 126 | 116 |
| chr19 | 38948083 | 38948323 | RYR1 | 0.53 | 248 | 252 |
| chr19 | 38948631 | 38948991 | RYR1 | 0.62 | 383 | 391 |
| chr19 | 38949761 | 38950001 | RYR1 | 0.62 | 97 | 83 |
| chr19 | 38950972 | 38951242 | RYR1 | 0.62 | 199 | 174 |
| chr19 | 38954054 | 38954174 | RYR1 | 0.65 | 44 | 35 |
| chr19 | 38954348 | 38954498 | RYR1 | 0.56 | 138 | 184 |
| chr19 | 38955260 | 38955380 | RYR1 | 0.63 | 11 | 19 |
| chr19 | 38956674 | 38957044 | RYR1 | 0.66 | 28 | 35 |
| chr19 | 38958200 | 38958470 | RYR1 | 0.60 | 140 | 179 |
| chr19 | 38959572 | 38959793 | RYR1 | 0.59 | 115 | 93 |
| chr19 | 38959939 | 38960168 | RYR1 | 0.60 | 195 | 231 |
| chr19 | 38963943 | 38964453 | RYR1 | 0.69 | 68 | 107 |
| chr19 | 38965903 | 38966143 | RYR1 | 0.61 | 49 | 61 |
| chr19 | 38968279 | 38968549 | RYR1 | 0.62 | 87 | 136 |
| chr19 | 38969007 | 38969277 | RYR1 | 0.56 | 105 | 93 |
| chr19 | 38973649 | 38973769 | RYR1 | 0.55 | 62 | 57 |
| chr19 | 38973922 | 38974162 | RYR1 | 0.67 | 10 | 11 |
| chr19 | 38976145 | 38976895 | RYR1 | 0.64 | 490 | 564 |
| chr19 | 38979764 | 38979884 | RYR1 | 0.50 | 60 | 54 |
| chr19 | 38980006 | 38980126 | RYR1 | 0.56 | 126 | 113 |
| chr19 | 38980665 | 38980935 | RYR1 | 0.60 | 121 | 122 |
| chr19 | 38981256 | 38981376 | RYR1 | 0.44 | 97 | 133 |
| chr19 | 38983101 | 38983322 | RYR1 | 0.59 | 111 | 92 |
| chr19 | 38984918 | 38985308 | RYR1 | 0.64 | 120 | 170 |
| chr19 | 38986791 | 38986971 | RYR1 | 0.62 | 90 | 101 |
| chr19 | 38986994 | 38987234 | RYR1 | 0.64 | 122 | 126 |
| chr19 | 38987456 | 38987606 | RYR1 | 0.62 | 44 | 59 |
| chr19 | 38989695 | 38989935 | RYR1 | 0.61 | 81 | 105 |
| chr19 | 38990217 | 38990487 | RYR1 | 0.68 | 47 | 42 |
| chr19 | 38990511 | 38990661 | RYR1 | 0.61 | 44 | 56 |
| chr19 | 38991155 | 38991665 | RYR1 | 0.65 | 123 | 174 |
| chr19 | 38993106 | 38993376 | RYR1 | 0.65 | 47 | 62 |
| chr19 | 38993504 | 38993624 | RYR1 | 0.63 | 48 | 54 |
| chr19 | 38994809 | 38995049 | RYR1 | 0.59 | 89 | 77 |
| chr19 | 38995349 | 38995589 | RYR1 | 0.58 | 139 | 138 |
| chr19 | 38995621 | 38995741 | RYR1 | 0.56 | 80 | 80 |
| chr19 | 38995933 | 38996053 | RYR1 | 0.54 | 37 | 24 |
| chr19 | 38996395 | 38996635 | RYR1 | 0.59 | 119 | 106 |
| chr19 | 38996919 | 38997039 | RYR1 | 0.59 | 99 | 122 |
| chr19 | 38997058 | 38997208 | RYR1 | 0.60 | 144 | 149 |
| chr19 | 38997410 | 38997650 | RYR1 | 0.63 | 44 | 66 |
| chr19 | 38998289 | 38998529 | RYR1 | 0.57 | 194 | 230 |
| chr19 | 39001081 | 39001480 | RYR1 | 0.56 | 342 | 415 |
| chr19 | 39002165 | 39002285 | RYR1 | 0.53 | 98 | 102 |
| chr19 | 39002681 | 39003183 | RYR1 | 0.64 | 238 | 252 |
| chr19 | 39005646 | 39005766 | RYR1 | 0.55 | 44 | 72 |
| chr19 | 39006671 | 39006911 | RYR1 | 0.66 | 0 | 14 |
| chr19 | 39007984 | 39008344 | RYR1 | 0.66 | 84 | 128 |
| chr19 | 39009763 | 39010153 | RYR1 | 0.67 | 147 | 176 |
| chr19 | 39013651 | 39013771 | RYR1 | 0.65 | 48 | 69 |
| chr19 | 39013812 | 39013962 | RYR1 | 0.53 | 262 | 289 |
| chr19 | 39014471 | 39014621 | RYR1 | 0.60 | 41 | 44 |
| chr19 | 39015923 | 39016176 | RYR1 | 0.64 | 86 | 76 |
| chr19 | 39017572 | 39017722 | RYR1 | 0.54 | 87 | 94 |
| chr19 | 39018235 | 39018475 | RYR1 | 0.66 | 53 | 67 |
| chr19 | 39018941 | 39019061 | RYR1 | 0.60 | 80 | 87 |
| chr19 | 39019196 | 39019346 | RYR1 | 0.57 | 36 | 60 |
| chr19 | 39019477 | 39019597 | RYR1 | 0.55 | 30 | 20 |
| chr19 | 39019638 | 39019758 | RYR1 | 0.65 | 47 | 33 |
| chr19 | 39023095 | 39023215 | RYR1 | 0.55 | 71 | 70 |
| chr19 | 39023253 | 39023403 | RYR1 | 0.62 | 68 | 59 |
| chr19 | 39025349 | 39025469 | RYR1 | 0.61 | 12 | 28 |
| chr19 | 39025730 | 39025880 | RYR1 | 0.56 | 78 | 72 |
| chr19 | 39025933 | 39026053 | RYR1 | 0.50 | 149 | 112 |
| chr19 | 39026613 | 39026733 | RYR1 | 0.55 | 26 | 19 |
| chr19 | 39027355 | 39027475 | RYR1 | 0.64 | 41 | 48 |
| chr19 | 39028469 | 39028619 | RYR1 | 0.56 | 146 | 177 |
| chr19 | 39033970 | 39034090 | RYR1 | 0.50 | 142 | 143 |
| chr19 | 39034115 | 39034355 | RYR1 | 0.62 | 121 | 139 |
| chr19 | 39034372 | 39034522 | RYR1 | 0.64 | 45 | 48 |
| chr19 | 39037065 | 39037185 | RYR1 | 0.55 | 80 | 88 |
| chr19 | 39038846 | 39039086 | RYR1 | 0.57 | 150 | 165 |
| chr19 | 39051713 | 39052103 | RYR1 | 0.62 | 171 | 208 |
| chr19 | 39055553 | 39055750 | RYR1 | 0.64 | 5 | 15 |
| chr19 | 39056031 | 39056462 | RYR1 | 0.73 | 2 | 6 |
| chr19 | 39057528 | 39057648 | RYR1 | 0.66 | 8 | 20 |
| chr19 | 39058364 | 39058604 | RYR1 | 0.61 | 143 | 132 |
| chr19 | 39061229 | 39061349 | RYR1 | 0.47 | 150 | 142 |
| chr19 | 39062604 | 39062929 | RYR1 | 0.59 | 199 | 226 |
| chr19 | 39063731 | 39064001 | RYR1 | 0.61 | 126 | 121 |
| chr19 | 39066519 | 39066639 | RYR1 | 0.50 | 95 | 115 |
| chr19 | 39068502 | 39068742 | RYR1 | 0.62 | 98 | 141 |
| chr19 | 39068753 | 39068873 | RYR1 | 0.60 | 36 | 39 |
| chr19 | 39070574 | 39070814 | RYR1 | 0.61 | 120 | 156 |
| chr19 | 39070926 | 39071162 | RYR1 | 0.62 | 77 | 75 |
| chr19 | 39075560 | 39075773 | RYR1 | 0.60 | 74 | 55 |
| chr19 | 39076519 | 39076669 | RYR1 | 0.58 | 94 | 113 |
| chr19 | 39076720 | 39076840 | RYR1 | 0.53 | 53 | 69 |
| chr19 | 39077100 | 39077250 | RYR1 | 0.45 | 104 | 130 |
| chr19 | 39077952 | 39078072 | RYR1 | 0.50 | 120 | 161 |
| chr1 | 26126859 | 26126979 | SEPN1 | 0.82 | 1 | 1 |
| chr1 | 26127442 | 26127712 | SEPN1 | 0.59 | 162 | 145 |
| chr1 | 26129146 | 26129266 | SEPN1 | 0.35 | 95 | 161 |
| chr1 | 26131549 | 26131819 | SEPN1 | 0.58 | 170 | 165 |
| chr1 | 26135025 | 26135295 | SEPN1 | 0.64 | 188 | 215 |
| chr1 | 26135428 | 26135679 | SEPN1 | 0.67 | 139 | 122 |
| chr1 | 26136092 | 26136362 | SEPN1 | 0.63 | 157 | 138 |
| chr1 | 26137895 | 26138045 | SEPN1 | 0.56 | 244 | 301 |
| chr1 | 26138155 | 26138395 | SEPN1 | 0.64 | 27 | 29 |
| chr1 | 26139140 | 26139290 | SEPN1 | 0.60 | 24 | 34 |
| chr1 | 26140105 | 26140225 | SEPN1 | 0.44 | 150 | 163 |
| chr1 | 26140305 | 26140678 | SEPN1 | 0.61 | 273 | 343 |
| chr1 | 26142003 | 26142243 | SEPN1 | 0.60 | 244 | 244 |
| chr19 | 55644245 | 55644365 | TNNT1 | 0.64 | 10 | 18 |
| chr19 | 55645184 | 55645334 | TNNT1 | 0.60 | 43 | 57 |
| chr19 | 55645382 | 55645583 | TNNT1 | 0.64 | 16 | 28 |
| chr19 | 55648435 | 55648585 | TNNT1 | 0.63 | 74 | 66 |
| chr19 | 55649295 | 55649445 | TNNT1 | 0.56 | 78 | 101 |
| chr19 | 55652199 | 55652349 | TNNT1 | 0.60 | 112 | 109 |
| chr19 | 55652491 | 55652731 | TNNT1 | 0.61 | 127 | 97 |
| chr19 | 55653171 | 55653321 | TNNT1 | 0.58 | 156 | 157 |
| chr19 | 55656832 | 55656982 | TNNT1 | 0.49 | 339 | 277 |
| chr19 | 55657757 | 55657877 | TNNT1 | 0.73 | 2 | 5 |
| chr19 | 55658001 | 55658121 | TNNT1 | 0.69 | 3 | 3 |
| chr19 | 55658322 | 55658442 | TNNT1 | 0.68 | 16 | 29 |
| chr19 | 55658449 | 55658569 | TNNT1 | 0.63 | 32 | 34 |
| chr9 | 35682028 | 35682178 | TPM2 | 0.60 | 58 | 55 |
| chr9 | 35682682 | 35682922 | TPM2 | 0.61 | 12 | 5 |
| chr9 | 35683136 | 35683256 | TPM2 | 0.58 | 2 | 6 |
| chr9 | 35684187 | 35684337 | TPM2 | 0.51 | 84 | 101 |
| chr9 | 35684425 | 35684575 | TPM2 | 0.49 | 121 | 108 |
| chr9 | 35684706 | 35684826 | TPM2 | 0.58 | 64 | 80 |
| chr9 | 35685008 | 35685158 | TPM2 | 0.63 | 125 | 105 |
| chr9 | 35685210 | 35685360 | TPM2 | 0.63 | 84 | 95 |
| chr9 | 35685369 | 35685830 | TPM2 | 0.60 | 257 | 314 |
| chr9 | 35688941 | 35689325 | TPM2 | 0.56 | 603 | 572 |
| chr9 | 35689644 | 35689884 | TPM2 | 0.56 | 191 | 226 |
| chr1 | 154130065 | 154130215 | TPM3 | 0.54 | 245 | 263 |
| chr1 | 154131394 | 154131544 | TPM3 | 0.52 | 166 | 149 |
| chr1 | 154140324 | 154140474 | TPM3 | 0.47 | 83 | 85 |
| chr1 | 154141759 | 154141879 | TPM3 | 0.50 | 49 | 63 |
| chr1 | 154142850 | 154142970 | TPM3 | 0.48 | 182 | 184 |
| chr1 | 154143065 | 154143215 | TPM3 | 0.32 | 369 | 346 |
| chr1 | 154143866 | 154143986 | TPM3 | 0.47 | 104 | 116 |
| chr1 | 154144452 | 154144891 | TPM3 | 0.45 | 849 | 996 |
| chr1 | 154145328 | 154145478 | TPM3 | 0.48 | 294 | 239 |
| chr1 | 154145498 | 154145738 | TPM3 | 0.44 | 282 | 252 |
| chr1 | 154148537 | 154148777 | TPM3 | 0.51 | 235 | 252 |
| chr1 | 154155409 | 154155649 | TPM3 | 0.65 | 88 | 123 |
| chr1 | 154163604 | 154163844 | TPM3 | 0.46 | 228 | 256 |
| chr1 | 154164315 | 154164555 | TPM3 | 0.49 | 202 | 187 |
| chr2 | 179391706 | 179392066 | TTN | 0.43 | 551 | 543 |
| chr2 | 179392143 | 179392503 | TTN | 0.45 | 610 | 579 |
| chr2 | 179392957 | 179393197 | TTN | 0.40 | 526 | 458 |
| chr2 | 179393240 | 179393960 | TTN | 0.41 | 1256 | 1263 |
| chr2 | 179394644 | 179394884 | TTN | 0.34 | 843 | 775 |
| chr2 | 179394951 | 179400591 | TTN | 0.44 | 13248 | 13185 |
| chr2 | 179400645 | 179401365 | TTN | 0.40 | 1400 | 1479 |
| chr2 | 179401637 | 179401997 | TTN | 0.47 | 452 | 451 |
| chr2 | 179402056 | 179402656 | TTN | 0.42 | 1004 | 926 |
| chr2 | 179403236 | 179403596 | TTN | 0.40 | 1001 | 999 |
| chr2 | 179403645 | 179404005 | TTN | 0.47 | 693 | 656 |
| chr2 | 179404040 | 179404760 | TTN | 0.43 | 2124 | 2020 |
| chr2 | 179404765 | 179405125 | TTN | 0.45 | 930 | 886 |
| chr2 | 179405983 | 179406339 | TTN | 0.47 | 482 | 420 |
| chr2 | 179406960 | 179407320 | TTN | 0.48 | 509 | 578 |
| chr2 | 179407352 | 179407712 | TTN | 0.42 | 1408 | 1425 |
| chr2 | 179407732 | 179408452 | TTN | 0.41 | 1770 | 1972 |
| chr2 | 179408521 | 179408881 | TTN | 0.42 | 1032 | 1117 |
| chr2 | 179408900 | 179409260 | TTN | 0.44 | 763 | 774 |
| chr2 | 179410087 | 179410447 | TTN | 0.47 | 1173 | 1254 |
| chr2 | 179410514 | 179410874 | TTN | 0.47 | 786 | 852 |
| chr2 | 179410903 | 179411263 | TTN | 0.45 | 1148 | 1166 |
| chr2 | 179411299 | 179411659 | TTN | 0.47 | 547 | 531 |
| chr2 | 179411700 | 179412060 | TTN | 0.42 | 1372 | 1472 |
| chr2 | 179412086 | 179414246 | TTN | 0.45 | 5284 | 5077 |
| chr2 | 179414266 | 179414626 | TTN | 0.41 | 995 | 981 |
| chr2 | 179414676 | 179415036 | TTN | 0.39 | 903 | 745 |
| chr2 | 179415660 | 179416020 | TTN | 0.43 | 454 | 409 |
| chr2 | 179416339 | 179418139 | TTN | 0.42 | 4341 | 4098 |
| chr2 | 179418201 | 179418561 | TTN | 0.43 | 1030 | 1057 |
| chr2 | 179418611 | 179418971 | TTN | 0.43 | 833 | 820 |
| chr2 | 179419149 | 179419509 | TTN | 0.45 | 802 | 880 |
| chr2 | 179419555 | 179419915 | TTN | 0.40 | 770 | 814 |
| chr2 | 179421542 | 179421902 | TTN | 0.44 | 804 | 767 |
| chr2 | 179421950 | 179423028 | TTN | 0.41 | 2939 | 2911 |
| chr2 | 179423035 | 179423395 | TTN | 0.41 | 781 | 696 |
| chr2 | 179424010 | 179441170 | TTN | 0.43 | 39647 | 39582 |
| chr2 | 179441226 | 179442656 | TTN | 0.41 | 3732 | 3755 |
| chr2 | 179442693 | 179442933 | TTN | 0.43 | 570 | 524 |
| chr2 | 179443269 | 179444186 | TTN | 0.42 | 2047 | 1933 |
| chr2 | 179444251 | 179444611 | TTN | 0.42 | 1143 | 1129 |
| chr2 | 179444630 | 179444990 | TTN | 0.43 | 1102 | 1157 |
| chr2 | 179445012 | 179445372 | TTN | 0.36 | 1108 | 1022 |
| chr2 | 179446198 | 179446558 | TTN | 0.45 | 716 | 725 |
| chr2 | 179446603 | 179446963 | TTN | 0.45 | 896 | 912 |
| chr2 | 179446990 | 179447350 | TTN | 0.43 | 664 | 639 |
| chr2 | 179447630 | 179447990 | TTN | 0.41 | 491 | 458 |
| chr2 | 179448303 | 179448663 | TTN | 0.44 | 407 | 385 |
| chr2 | 179448973 | 179449333 | TTN | 0.44 | 937 | 921 |
| chr2 | 179449365 | 179449725 | TTN | 0.49 | 1138 | 1161 |
| chr2 | 179449756 | 179450116 | TTN | 0.40 | 1453 | 1380 |
| chr2 | 179451202 | 179451562 | TTN | 0.44 | 913 | 956 |
| chr2 | 179451814 | 179452174 | TTN | 0.44 | 680 | 775 |
| chr2 | 179452204 | 179452564 | TTN | 0.43 | 586 | 560 |
| chr2 | 179452605 | 179452965 | TTN | 0.45 | 307 | 339 |
| chr2 | 179453247 | 179456247 | TTN | 0.43 | 8232 | 8095 |
| chr2 | 179456292 | 179456652 | TTN | 0.41 | 1144 | 1117 |
| chr2 | 179456674 | 179457034 | TTN | 0.42 | 572 | 598 |
| chr2 | 179457066 | 179457426 | TTN | 0.37 | 1073 | 1026 |
| chr2 | 179457475 | 179457835 | TTN | 0.43 | 917 | 926 |
| chr2 | 179457870 | 179458230 | TTN | 0.39 | 1114 | 1104 |
| chr2 | 179458264 | 179458624 | TTN | 0.38 | 1076 | 1010 |
| chr2 | 179458648 | 179459008 | TTN | 0.42 | 1035 | 1064 |
| chr2 | 179459041 | 179459401 | TTN | 0.39 | 934 | 871 |
| chr2 | 179460204 | 179460564 | TTN | 0.41 | 392 | 365 |
| chr2 | 179462224 | 179462585 | TTN | 0.43 | 796 | 792 |
| chr2 | 179462589 | 179462829 | TTN | 0.37 | 768 | 771 |
| chr2 | 179463186 | 179463426 | TTN | 0.41 | 727 | 740 |
| chr2 | 179463451 | 179463811 | TTN | 0.46 | 952 | 957 |
| chr2 | 179463842 | 179464202 | TTN | 0.41 | 1207 | 1249 |
| chr2 | 179464248 | 179464608 | TTN | 0.40 | 1262 | 1168 |
| chr2 | 179465467 | 179465919 | TTN | 0.45 | 1020 | 1077 |
| chr2 | 179465961 | 179466321 | TTN | 0.43 | 865 | 853 |
| chr2 | 179466329 | 179466922 | TTN | 0.36 | 1607 | 1673 |
| chr2 | 179466982 | 179467342 | TTN | 0.41 | 786 | 799 |
| chr2 | 179468577 | 179469057 | TTN | 0.45 | 1189 | 1128 |
| chr2 | 179469409 | 179469649 | TTN | 0.41 | 728 | 672 |
| chr2 | 179469687 | 179470047 | TTN | 0.45 | 856 | 799 |
| chr2 | 179470110 | 179470470 | TTN | 0.41 | 704 | 755 |
| chr2 | 179471714 | 179472074 | TTN | 0.39 | 960 | 997 |
| chr2 | 179472089 | 179472449 | TTN | 0.43 | 1163 | 1143 |
| chr2 | 179472479 | 179472839 | TTN | 0.46 | 906 | 887 |
| chr2 | 179472874 | 179473234 | TTN | 0.41 | 475 | 437 |
| chr2 | 179473303 | 179473663 | TTN | 0.40 | 641 | 671 |
| chr2 | 179473875 | 179474355 | TTN | 0.39 | 722 | 727 |
| chr2 | 179474381 | 179474741 | TTN | 0.45 | 1294 | 1406 |
| chr2 | 179474786 | 179475146 | TTN | 0.40 | 1073 | 1004 |
| chr2 | 179475678 | 179476038 | TTN | 0.39 | 936 | 951 |
| chr2 | 179476071 | 179476431 | TTN | 0.43 | 1013 | 977 |
| chr2 | 179476462 | 179476702 | TTN | 0.43 | 774 | 778 |
| chr2 | 179476716 | 179476956 | TTN | 0.38 | 482 | 503 |
| chr2 | 179476973 | 179477333 | TTN | 0.45 | 582 | 600 |
| chr2 | 179477469 | 179478065 | TTN | 0.42 | 1238 | 1182 |
| chr2 | 179478450 | 179478690 | TTN | 0.44 | 716 | 707 |
| chr2 | 179478746 | 179479106 | TTN | 0.42 | 500 | 483 |
| chr2 | 179479156 | 179479516 | TTN | 0.42 | 846 | 812 |
| chr2 | 179479547 | 179479754 | TTN | 0.44 | 165 | 174 |
| chr2 | 179480002 | 179480242 | TTN | 0.41 | 715 | 713 |
| chr2 | 179480321 | 179480561 | TTN | 0.37 | 593 | 618 |
| chr2 | 179481161 | 179481401 | TTN | 0.42 | 615 | 544 |
| chr2 | 179481417 | 179481777 | TTN | 0.40 | 1069 | 1094 |
| chr2 | 179481783 | 179482023 | TTN | 0.35 | 563 | 545 |
| chr2 | 179482025 | 179482265 | TTN | 0.40 | 279 | 296 |
| chr2 | 179482476 | 179482836 | TTN | 0.44 | 903 | 913 |
| chr2 | 179482886 | 179483246 | TTN | 0.42 | 1366 | 1241 |
| chr2 | 179483265 | 179483625 | TTN | 0.39 | 987 | 873 |
| chr2 | 179484300 | 179484896 | TTN | 0.36 | 1674 | 1542 |
| chr2 | 179484907 | 179485387 | TTN | 0.40 | 1391 | 1222 |
| chr2 | 179485400 | 179485760 | TTN | 0.37 | 1335 | 1235 |
| chr2 | 179485781 | 179486141 | TTN | 0.39 | 925 | 901 |
| chr2 | 179486154 | 179486514 | TTN | 0.40 | 1141 | 1116 |
| chr2 | 179486530 | 179486770 | TTN | 0.36 | 650 | 759 |
| chr2 | 179487385 | 179487505 | TTN | 0.42 | 85 | 106 |
| chr2 | 179489144 | 179489504 | TTN | 0.41 | 973 | 949 |
| chr2 | 179489941 | 179490181 | TTN | 0.37 | 867 | 876 |
| chr2 | 179493978 | 179494218 | TTN | 0.47 | 418 | 417 |
| chr2 | 179494910 | 179495150 | TTN | 0.36 | 393 | 395 |
| chr2 | 179495480 | 179496073 | TTN | 0.42 | 1307 | 1318 |
| chr2 | 179496826 | 179497186 | TTN | 0.38 | 448 | 405 |
| chr2 | 179497205 | 179497565 | TTN | 0.39 | 1053 | 1049 |
| chr2 | 179497587 | 179497827 | TTN | 0.41 | 480 | 472 |
| chr2 | 179497863 | 179498451 | TTN | 0.41 | 1189 | 1168 |
| chr2 | 179498496 | 179498856 | TTN | 0.39 | 1094 | 1051 |
| chr2 | 179499044 | 179499632 | TTN | 0.41 | 1398 | 1417 |
| chr2 | 179499841 | 179500081 | TTN | 0.39 | 656 | 638 |
| chr2 | 179500124 | 179500484 | TTN | 0.36 | 637 | 604 |
| chr2 | 179500648 | 179501008 | TTN | 0.44 | 804 | 801 |
| chr2 | 179501085 | 179501565 | TTN | 0.41 | 925 | 863 |
| chr2 | 179502060 | 179502180 | TTN | 0.36 | 169 | 170 |
| chr2 | 179504410 | 179504530 | TTN | 0.49 | 224 | 211 |
| chr2 | 179504745 | 179504865 | TTN | 0.48 | 241 | 261 |
| chr2 | 179505252 | 179505372 | TTN | 0.39 | 363 | 298 |
| chr2 | 179505944 | 179506064 | TTN | 0.31 | 53 | 56 |
| chr2 | 179506941 | 179507063 | TTN | 0.37 | 299 | 313 |
| chr2 | 179509248 | 179509368 | TTN | 0.34 | 195 | 184 |
| chr2 | 179510581 | 179510821 | TTN | 0.38 | 380 | 353 |
| chr2 | 179511188 | 179511308 | TTN | 0.36 | 205 | 210 |
| chr2 | 179511789 | 179511909 | TTN | 0.30 | 143 | 134 |
| chr2 | 179512089 | 179512209 | TTN | 0.45 | 261 | 232 |
| chr2 | 179513956 | 179514076 | TTN | 0.39 | 380 | 399 |
| chr2 | 179514259 | 179514379 | TTN | 0.41 | 297 | 324 |
| chr2 | 179514522 | 179514642 | TTN | 0.40 | 367 | 332 |
| chr2 | 179514824 | 179515064 | TTN | 0.37 | 296 | 281 |
| chr2 | 179515459 | 179515581 | TTN | 0.47 | 230 | 190 |
| chr2 | 179515948 | 179516068 | TTN | 0.37 | 490 | 522 |
| chr2 | 179516161 | 179516281 | TTN | 0.49 | 288 | 282 |
| chr2 | 179516374 | 179516494 | TTN | 0.45 | 222 | 214 |
| chr2 | 179516592 | 179516712 | TTN | 0.55 | 142 | 124 |
| chr2 | 179516804 | 179516924 | TTN | 0.43 | 639 | 698 |
| chr2 | 179516972 | 179517092 | TTN | 0.48 | 778 | 825 |
| chr2 | 179517166 | 179517286 | TTN | 0.41 | 588 | 523 |
| chr2 | 179517343 | 179517463 | TTN | 0.45 | 609 | 555 |
| chr2 | 179517533 | 179517653 | TTN | 0.46 | 471 | 494 |
| chr2 | 179517950 | 179518066 | TTN | 0.43 | 490 | 467 |
| chr2 | 179518133 | 179518253 | TTN | 0.40 | 173 | 154 |
| chr2 | 179518350 | 179518470 | TTN | 0.43 | 24 | 26 |
| chr2 | 179518523 | 179518643 | TTN | 0.45 | 98 | 76 |
| chr2 | 179518720 | 179518840 | TTN | 0.44 | 305 | 302 |
| chr2 | 179518916 | 179519036 | TTN | 0.41 | 383 | 423 |
| chr2 | 179519156 | 179519276 | TTN | 0.49 | 30 | 31 |
| chr2 | 179519453 | 179519573 | TTN | 0.47 | 12 | 9 |
| chr2 | 179519620 | 179519740 | TTN | 0.47 | 9 | 5 |
| chr2 | 179522206 | 179522326 | TTN | 0.40 | 211 | 203 |
| chr2 | 179522393 | 179522513 | TTN | 0.40 | 49 | 33 |
| chr2 | 179522610 | 179522730 | TTN | 0.43 | 64 | 65 |
| chr2 | 179522783 | 179522903 | TTN | 0.46 | 224 | 257 |
| chr2 | 179522980 | 179523100 | TTN | 0.44 | 23 | 30 |
| chr2 | 179523176 | 179523296 | TTN | 0.42 | 2 | 0 |
| chr2 | 179523416 | 179523536 | TTN | 0.49 | 79 | 97 |
| chr2 | 179523713 | 179523833 | TTN | 0.47 | 16 | 12 |
| chr2 | 179523880 | 179524000 | TTN | 0.47 | 12 | 6 |
| chr2 | 179526467 | 179526587 | TTN | 0.41 | 235 | 204 |
| chr2 | 179526654 | 179526774 | TTN | 0.40 | 40 | 28 |
| chr2 | 179526871 | 179526991 | TTN | 0.43 | 50 | 60 |
| chr2 | 179527044 | 179527164 | TTN | 0.45 | 195 | 190 |
| chr2 | 179527241 | 179527361 | TTN | 0.44 | 43 | 46 |
| chr2 | 179527437 | 179527557 | TTN | 0.42 | 33 | 24 |
| chr2 | 179527677 | 179527797 | TTN | 0.49 | 85 | 135 |
| chr2 | 179527974 | 179528094 | TTN | 0.46 | 364 | 323 |
| chr2 | 179528141 | 179528261 | TTN | 0.47 | 401 | 339 |
| chr2 | 179528335 | 179528455 | TTN | 0.42 | 693 | 642 |
| chr2 | 179528527 | 179528647 | TTN | 0.47 | 515 | 553 |
| chr2 | 179528723 | 179528843 | TTN | 0.41 | 298 | 355 |
| chr2 | 179529165 | 179529285 | TTN | 0.41 | 356 | 352 |
| chr2 | 179529362 | 179529482 | TTN | 0.42 | 227 | 235 |
| chr2 | 179529555 | 179529675 | TTN | 0.44 | 185 | 193 |
| chr2 | 179530085 | 179530205 | TTN | 0.37 | 450 | 491 |
| chr2 | 179531529 | 179531649 | TTN | 0.35 | 188 | 174 |
| chr2 | 179531944 | 179532064 | TTN | 0.41 | 200 | 163 |
| chr2 | 179532150 | 179532270 | TTN | 0.34 | 298 | 257 |
| chr2 | 179532346 | 179532466 | TTN | 0.33 | 496 | 489 |
| chr2 | 179534076 | 179534196 | TTN | 0.31 | 374 | 401 |
| chr2 | 179534300 | 179534420 | TTN | 0.38 | 660 | 514 |
| chr2 | 179534916 | 179535043 | TTN | 0.37 | 342 | 303 |
| chr2 | 179535796 | 179535916 | TTN | 0.38 | 176 | 156 |
| chr2 | 179536661 | 179536875 | TTN | 0.32 | 525 | 589 |
| chr2 | 179536952 | 179537072 | TTN | 0.30 | 366 | 313 |
| chr2 | 179537110 | 179537230 | TTN | 0.38 | 615 | 602 |
| chr2 | 179537335 | 179537455 | TTN | 0.37 | 354 | 319 |
| chr2 | 179538338 | 179538458 | TTN | 0.37 | 205 | 233 |
| chr2 | 179539016 | 179539148 | TTN | 0.43 | 264 | 271 |
| chr2 | 179539742 | 179539862 | TTN | 0.36 | 156 | 140 |
| chr2 | 179540624 | 179540744 | TTN | 0.40 | 311 | 280 |
| chr2 | 179542315 | 179542675 | TTN | 0.43 | 1089 | 1044 |
| chr2 | 179542833 | 179542953 | TTN | 0.41 | 492 | 480 |
| chr2 | 179543122 | 179543242 | TTN | 0.45 | 220 | 175 |
| chr2 | 179543455 | 179543575 | TTN | 0.44 | 360 | 321 |
| chr2 | 179544044 | 179544164 | TTN | 0.41 | 251 | 233 |
| chr2 | 179544307 | 179544427 | TTN | 0.44 | 99 | 98 |
| chr2 | 179544581 | 179544821 | TTN | 0.41 | 377 | 404 |
| chr2 | 179544956 | 179545079 | TTN | 0.43 | 376 | 374 |
| chr2 | 179545791 | 179545911 | TTN | 0.35 | 106 | 56 |
| chr2 | 179546079 | 179546199 | TTN | 0.39 | 47 | 33 |
| chr2 | 179546366 | 179546486 | TTN | 0.43 | 199 | 212 |
| chr2 | 179547406 | 179547646 | TTN | 0.35 | 480 | 459 |
| chr2 | 179547917 | 179548037 | TTN | 0.31 | 14 | 7 |
| chr2 | 179548707 | 179548827 | TTN | 0.40 | 134 | 117 |
| chr2 | 179549038 | 179549158 | TTN | 0.40 | 57 | 47 |
| chr2 | 179549374 | 179549494 | TTN | 0.45 | 116 | 85 |
| chr2 | 179549614 | 179549734 | TTN | 0.37 | 43 | 67 |
| chr2 | 179549958 | 179550078 | TTN | 0.35 | 9 | 14 |
| chr2 | 179550224 | 179550344 | TTN | 0.40 | 59 | 49 |
| chr2 | 179552834 | 179552954 | TTN | 0.42 | 215 | 183 |
| chr2 | 179553394 | 179553514 | TTN | 0.39 | 123 | 130 |
| chr2 | 179553761 | 179553881 | TTN | 0.52 | 264 | 247 |
| chr2 | 179553998 | 179554118 | TTN | 0.41 | 363 | 319 |
| chr2 | 179554221 | 179554341 | TTN | 0.40 | 263 | 181 |
| chr2 | 179554521 | 179554641 | TTN | 0.45 | 400 | 412 |
| chr2 | 179556724 | 179556844 | TTN | 0.49 | 227 | 211 |
| chr2 | 179557205 | 179557325 | TTN | 0.45 | 25 | 31 |
| chr2 | 179558315 | 179558435 | TTN | 0.35 | 299 | 333 |
| chr2 | 179558631 | 179558751 | TTN | 0.40 | 151 | 143 |
| chr2 | 179559304 | 179559424 | TTN | 0.35 | 162 | 107 |
| chr2 | 179559534 | 179559654 | TTN | 0.31 | 164 | 115 |
| chr2 | 179560045 | 179560165 | TTN | 0.34 | 208 | 169 |
| chr2 | 179560518 | 179560638 | TTN | 0.32 | 190 | 187 |
| chr2 | 179560784 | 179561015 | TTN | 0.36 | 341 | 309 |
| chr2 | 179561811 | 179561931 | TTN | 0.30 | 31 | 44 |
| chr2 | 179563522 | 179563665 | TTN | 0.31 | 153 | 192 |
| chr2 | 179565828 | 179565948 | TTN | 0.44 | 361 | 355 |
| chr2 | 179566224 | 179566344 | TTN | 0.47 | 397 | 404 |
| chr2 | 179566719 | 179566839 | TTN | 0.29 | 238 | 242 |
| chr2 | 179566873 | 179566993 | TTN | 0.53 | 200 | 197 |
| chr2 | 179567165 | 179567405 | TTN | 0.44 | 665 | 580 |
| chr2 | 179568823 | 179569183 | TTN | 0.43 | 942 | 1021 |
| chr2 | 179569190 | 179569550 | TTN | 0.35 | 753 | 746 |
| chr2 | 179569588 | 179569708 | TTN | 0.36 | 413 | 465 |
| chr2 | 179569872 | 179570112 | TTN | 0.36 | 365 | 327 |
| chr2 | 179571152 | 179571503 | TTN | 0.39 | 919 | 940 |
| chr2 | 179571574 | 179571694 | TTN | 0.44 | 182 | 193 |
| chr2 | 179572216 | 179572576 | TTN | 0.45 | 560 | 585 |
| chr2 | 179574257 | 179574617 | TTN | 0.43 | 690 | 678 |
| chr2 | 179575325 | 179575685 | TTN | 0.47 | 640 | 676 |
| chr2 | 179575752 | 179576112 | TTN | 0.39 | 944 | 945 |
| chr2 | 179576629 | 179576989 | TTN | 0.40 | 780 | 826 |
| chr2 | 179577000 | 179577360 | TTN | 0.38 | 1080 | 1091 |
| chr2 | 179577382 | 179577742 | TTN | 0.39 | 676 | 714 |
| chr2 | 179577774 | 179578107 | TTN | 0.41 | 783 | 869 |
| chr2 | 179578582 | 179578942 | TTN | 0.41 | 582 | 594 |
| chr2 | 179578979 | 179579339 | TTN | 0.42 | 820 | 833 |
| chr2 | 179579671 | 179580031 | TTN | 0.43 | 999 | 1078 |
| chr2 | 179580180 | 179580540 | TTN | 0.45 | 515 | 576 |
| chr2 | 179581785 | 179582145 | TTN | 0.43 | 597 | 555 |
| chr2 | 179582213 | 179582573 | TTN | 0.42 | 775 | 714 |
| chr2 | 179582628 | 179582988 | TTN | 0.42 | 1146 | 1195 |
| chr2 | 179583007 | 179583367 | TTN | 0.42 | 956 | 1006 |
| chr2 | 179583380 | 179583740 | TTN | 0.45 | 533 | 513 |
| chr2 | 179583854 | 179584214 | TTN | 0.50 | 715 | 807 |
| chr2 | 179584239 | 179584599 | TTN | 0.39 | 1086 | 1044 |
| chr2 | 179584670 | 179585030 | TTN | 0.42 | 655 | 575 |
| chr2 | 179585070 | 179585430 | TTN | 0.40 | 752 | 729 |
| chr2 | 179585608 | 179585968 | TTN | 0.40 | 630 | 671 |
| chr2 | 179586537 | 179586897 | TTN | 0.40 | 1363 | 1304 |
| chr2 | 179586949 | 179587309 | TTN | 0.42 | 1128 | 1121 |
| chr2 | 179587344 | 179587704 | TTN | 0.38 | 758 | 799 |
| chr2 | 179587731 | 179588091 | TTN | 0.38 | 899 | 946 |
| chr2 | 179588103 | 179588463 | TTN | 0.42 | 939 | 865 |
| chr2 | 179588546 | 179588906 | TTN | 0.43 | 1067 | 1024 |
| chr2 | 179588945 | 179589305 | TTN | 0.45 | 741 | 832 |
| chr2 | 179590064 | 179590415 | TTN | 0.43 | 818 | 901 |
| chr2 | 179590453 | 179590813 | TTN | 0.40 | 736 | 728 |
| chr2 | 179591777 | 179592137 | TTN | 0.42 | 708 | 687 |
| chr2 | 179592270 | 179592630 | TTN | 0.39 | 1172 | 1184 |
| chr2 | 179592800 | 179593160 | TTN | 0.38 | 401 | 348 |
| chr2 | 179593185 | 179593545 | TTN | 0.40 | 607 | 609 |
| chr2 | 179593576 | 179593936 | TTN | 0.38 | 776 | 765 |
| chr2 | 179593973 | 179594333 | TTN | 0.44 | 865 | 881 |
| chr2 | 179594351 | 179594711 | TTN | 0.42 | 623 | 638 |
| chr2 | 179594778 | 179595138 | TTN | 0.44 | 628 | 621 |
| chr2 | 179595195 | 179595555 | TTN | 0.39 | 998 | 931 |
| chr2 | 179595610 | 179595970 | TTN | 0.41 | 1394 | 1308 |
| chr2 | 179595990 | 179596350 | TTN | 0.44 | 1017 | 941 |
| chr2 | 179596378 | 179596738 | TTN | 0.46 | 808 | 937 |
| chr2 | 179596753 | 179597113 | TTN | 0.42 | 1118 | 1103 |
| chr2 | 179597125 | 179597485 | TTN | 0.43 | 751 | 707 |
| chr2 | 179597524 | 179597884 | TTN | 0.46 | 629 | 647 |
| chr2 | 179597924 | 179598284 | TTN | 0.43 | 892 | 1048 |
| chr2 | 179598299 | 179598659 | TTN | 0.40 | 1222 | 1252 |
| chr2 | 179599013 | 179599373 | TTN | 0.38 | 1283 | 1270 |
| chr2 | 179599394 | 179599754 | TTN | 0.40 | 670 | 626 |
| chr2 | 179600219 | 179600819 | TTN | 0.43 | 1101 | 1118 |
| chr2 | 179602767 | 179603127 | TTN | 0.47 | 556 | 576 |
| chr2 | 179603817 | 179606664 | TTN | 0.43 | 7120 | 7110 |
| chr2 | 179610298 | 179616778 | TTN | 0.38 | 7785 | 7333 |
| chr2 | 179617818 | 179617938 | TTN | 0.29 | 136 | 125 |
| chr2 | 179620936 | 179621536 | TTN | 0.42 | 1305 | 1400 |
| chr2 | 179622215 | 179622695 | TTN | 0.44 | 1194 | 1141 |
| chr2 | 179623684 | 179623924 | TTN | 0.41 | 249 | 245 |
| chr2 | 179628846 | 179629086 | TTN | 0.44 | 234 | 230 |
| chr2 | 179629185 | 179629575 | TTN | 0.49 | 495 | 445 |
| chr2 | 179631103 | 179631343 | TTN | 0.42 | 274 | 253 |
| chr2 | 179632448 | 179632688 | TTN | 0.42 | 336 | 324 |
| chr2 | 179632691 | 179632910 | TTN | 0.40 | 247 | 249 |
| chr2 | 179633349 | 179633709 | TTN | 0.39 | 238 | 252 |
| chr2 | 179634445 | 179634715 | TTN | 0.40 | 457 | 412 |
| chr2 | 179634736 | 179635450 | TTN | 0.43 | 974 | 1002 |
| chr2 | 179635473 | 179635593 | TTN | 0.40 | 485 | 489 |
| chr2 | 179635887 | 179636247 | TTN | 0.43 | 252 | 273 |
| chr2 | 179637785 | 179638500 | TTN | 0.36 | 716 | 710 |
| chr2 | 179638520 | 179638880 | TTN | 0.42 | 448 | 400 |
| chr2 | 179638886 | 179639246 | TTN | 0.39 | 369 | 394 |
| chr2 | 179639608 | 179639968 | TTN | 0.39 | 362 | 336 |
| chr2 | 179640029 | 179641829 | TTN | 0.45 | 2042 | 2279 |
| chr2 | 179641839 | 179642079 | TTN | 0.37 | 330 | 394 |
| chr2 | 179642108 | 179642348 | TTN | 0.34 | 246 | 190 |
| chr2 | 179642386 | 179642746 | TTN | 0.43 | 340 | 250 |
| chr2 | 179643542 | 179643902 | TTN | 0.37 | 293 | 236 |
| chr2 | 179643952 | 179644222 | TTN | 0.32 | 308 | 231 |
| chr2 | 179644672 | 179644792 | TTN | 0.32 | 236 | 199 |
| chr2 | 179644819 | 179645000 | TTN | 0.36 | 293 | 274 |
| chr2 | 179645798 | 179646038 | TTN | 0.36 | 189 | 144 |
| chr2 | 179646926 | 179647166 | TTN | 0.49 | 166 | 164 |
| chr2 | 179647237 | 179647357 | TTN | 0.33 | 59 | 96 |
| chr2 | 179647481 | 179647841 | TTN | 0.46 | 233 | 226 |
| chr2 | 179648419 | 179648539 | TTN | 0.37 | 203 | 214 |
| chr2 | 179648757 | 179649117 | TTN | 0.50 | 240 | 296 |
| chr2 | 179650287 | 179650527 | TTN | 0.39 | 215 | 199 |
| chr2 | 179650541 | 179650901 | TTN | 0.49 | 211 | 258 |
| chr2 | 179654035 | 179654275 | TTN | 0.37 | 324 | 268 |
| chr2 | 179654653 | 179654893 | TTN | 0.32 | 282 | 331 |
| chr2 | 179655383 | 179655623 | TTN | 0.39 | 265 | 272 |
| chr2 | 179656741 | 179656981 | TTN | 0.30 | 231 | 216 |
| chr2 | 179658079 | 179658319 | TTN | 0.39 | 250 | 275 |
| chr2 | 179659051 | 179659321 | TTN | 0.50 | 379 | 402 |
| chr2 | 179659603 | 179659993 | TTN | 0.53 | 223 | 249 |
| chr2 | 179664132 | 179664515 | TTN | 0.52 | 213 | 239 |
| chr2 | 179664534 | 179664654 | TTN | 0.38 | 140 | 104 |
| chr2 | 179665085 | 179665445 | TTN | 0.51 | 421 | 408 |
| chr2 | 179666846 | 179667086 | TTN | 0.54 | 180 | 200 |
| chr2 | 179669263 | 179669383 | TTN | 0.50 | 86 | 82 |
| chr2 | 179682208 | 179682328 | TTN | 0.40 | 92 | 112 |
