# Supplementary material for: An Integrated Diagnosis Strategy for Congenital Myopathies
Source: PLoS One. 2013 Jun 24;8(6):e67527. doi: 10.1371/journal.pone.0067527 (PMC3691193; doi:10.1371/journal.pone.0067527)
Supplement: Table S4 — Coverage of the congenital myopathy genes for families 3-6. (DOCX) [file pone.0067527.s006.docx]

**Supplementary Table 4:** Coverage of the congenital myopathy genes for families 3-6

| **Chr** | **Start** | **End** | **Gene** | **GC (%)** | **Read number AKY21** | **Read number IM26** | **Read number AHY58** | **Read number AGT66** | **Read number AGT67** | **Read number AHE6** |
| --- | --- | --- | --- | --- | --- | --- | --- | --- | --- | --- |
| chr1 | 229567197 | 229567437 | ACTA1 | 0.62 | 99 | 106 | 136 | 103 | 128 | 37 |
| chr1 | 229567438 | 229567678 | ACTA1 | 0.62 | 91 | 97 | 112 | 94 | 104 | 39 |
| chr1 | 229567722 | 229567956 | ACTA1 | 0.65 | 48 | 53 | 78 | 60 | 51 | 21 |
| chr1 | 229568014 | 229568217 | ACTA1 | 0.66 | 49 | 53 | 70 | 52 | 64 | 21 |
| chr1 | 229568289 | 229568644 | ACTA1 | 0.61 | 206 | 320 | 259 | 209 | 304 | 91 |
| chr1 | 229568677 | 229568917 | ACTA1 | 0.68 | 40 | 45 | 47 | 37 | 33 | 40 |
| chr2 | 127806095 | 127806215 | BIN1 | 0.62 | 73 | 95 | 126 | 114 | 118 | 44 |
| chr2 | 127807987 | 127808107 | BIN1 | 0.61 | 85 | 150 | 148 | 89 | 129 | 36 |
| chr2 | 127808372 | 127808492 | BIN1 | 0.64 | 59 | 60 | 86 | 96 | 70 | 21 |
| chr2 | 127808714 | 127808834 | BIN1 | 0.66 | 14 | 25 | 23 | 22 | 16 | 6 |
| chr2 | 127809824 | 127809944 | BIN1 | 0.70 | 9 | 8 | 10 | 10 | 11 | 4 |
| chr2 | 127810949 | 127811069 | BIN1 | 0.58 | 31 | 36 | 50 | 25 | 45 | 3 |
| chr2 | 127811474 | 127811594 | BIN1 | 0.66 | 2 | 3 | 11 | 2 | 4 | 3 |
| chr2 | 127814992 | 127815232 | BIN1 | 0.68 | 6 | 9 | 20 | 6 | 9 | 1 |
| chr2 | 127816489 | 127816609 | BIN1 | 0.60 | 7 | 5 | 11 | 6 | 5 | 1 |
| chr2 | 127816638 | 127816758 | BIN1 | 0.63 | 8 | 10 | 14 | 7 | 12 | 5 |
| chr2 | 127818133 | 127818253 | BIN1 | 0.60 | 14 | 11 | 27 | 15 | 11 | 6 |
| chr2 | 127819671 | 127819791 | BIN1 | 0.61 | 19 | 41 | 31 | 18 | 44 | 14 |
| chr2 | 127821124 | 127821244 | BIN1 | 0.58 | 31 | 40 | 51 | 32 | 44 | 14 |
| chr2 | 127821491 | 127821611 | BIN1 | 0.63 | 39 | 57 | 69 | 40 | 55 | 16 |
| chr2 | 127825724 | 127825844 | BIN1 | 0.59 | 27 | 55 | 66 | 31 | 51 | 15 |
| chr2 | 127826493 | 127826613 | BIN1 | 0.57 | 54 | 83 | 80 | 80 | 93 | 29 |
| chr2 | 127827558 | 127827678 | BIN1 | 0.59 | 54 | 80 | 81 | 100 | 95 | 22 |
| chr2 | 127828115 | 127828235 | BIN1 | 0.58 | 196 | 270 | 206 | 259 | 299 | 89 |
| chr2 | 127828304 | 127828424 | BIN1 | 0.64 | 77 | 68 | 35 | 73 | 45 | 48 |
| chr2 | 127834181 | 127834301 | BIN1 | 0.55 | 94 | 131 | 126 | 105 | 123 | 25 |
| chr2 | 127864417 | 127864537 | BIN1 | 0.68 | 21 | 26 | 53 | 22 | 32 | 15 |
| chr14 | 35182066 | 35182186 | CFL2 | 0.37 | 197 | 327 | 178 | 269 | 245 | 74 |
| chr14 | 35182243 | 35182363 | CFL2 | 0.32 | 121 | 102 | 34 | 100 | 35 | 83 |
| chr14 | 35182433 | 35182793 | CFL2 | 0.36 | 619 | 693 | 395 | 575 | 505 | 307 |
| chr14 | 35182820 | 35182940 | CFL2 | 0.39 | 191 | 234 | 177 | 196 | 193 | 112 |
| chr14 | 35183684 | 35183804 | CFL2 | 0.74 | 10 | 14 | 14 | 17 | 9 | 1 |
| chr12 | 41302204 | 41302324 | CNTN1 | 0.32 | 120 | 219 | 132 | 138 | 146 | 54 |
| chr12 | 41303830 | 41303950 | CNTN1 | 0.31 | 177 | 231 | 196 | 185 | 215 | 84 |
| chr12 | 41312386 | 41312626 | CNTN1 | 0.39 | 402 | 436 | 250 | 405 | 335 | 146 |
| chr12 | 41316023 | 41316263 | CNTN1 | 0.39 | 349 | 462 | 233 | 403 | 316 | 134 |
| chr12 | 41318346 | 41318466 | CNTN1 | 0.44 | 119 | 182 | 110 | 113 | 134 | 42 |
| chr12 | 41323580 | 41323820 | CNTN1 | 0.38 | 478 | 561 | 298 | 481 | 420 | 168 |
| chr12 | 41327252 | 41327372 | CNTN1 | 0.35 | 319 | 479 | 185 | 343 | 357 | 93 |
| chr12 | 41327469 | 41327709 | CNTN1 | 0.37 | 248 | 243 | 168 | 209 | 124 | 97 |
| chr12 | 41330524 | 41330764 | CNTN1 | 0.44 | 406 | 586 | 312 | 490 | 463 | 177 |
| chr12 | 41331310 | 41331550 | CNTN1 | 0.31 | 275 | 330 | 240 | 303 | 294 | 107 |
| chr12 | 41333091 | 41333331 | CNTN1 | 0.38 | 278 | 369 | 166 | 313 | 263 | 105 |
| chr12 | 41337342 | 41337582 | CNTN1 | 0.31 | 320 | 365 | 246 | 327 | 250 | 109 |
| chr12 | 41337764 | 41338004 | CNTN1 | 0.37 | 338 | 415 | 246 | 379 | 385 | 149 |
| chr12 | 41352855 | 41353095 | CNTN1 | 0.37 | 252 | 369 | 203 | 310 | 263 | 108 |
| chr12 | 41374669 | 41374909 | CNTN1 | 0.41 | 273 | 317 | 167 | 241 | 258 | 106 |
| chr12 | 41386876 | 41387116 | CNTN1 | 0.36 | 554 | 570 | 216 | 519 | 376 | 159 |
| chr12 | 41408004 | 41408124 | CNTN1 | 0.43 | 215 | 260 | 87 | 221 | 212 | 63 |
| chr12 | 41410480 | 41410720 | CNTN1 | 0.40 | 432 | 482 | 273 | 408 | 392 | 158 |
| chr12 | 41414130 | 41414250 | CNTN1 | 0.37 | 141 | 205 | 109 | 150 | 143 | 59 |
| chr12 | 41418924 | 41419164 | CNTN1 | 0.47 | 98 | 121 | 53 | 114 | 115 | 40 |
| chr12 | 41421654 | 41421774 | CNTN1 | 0.45 | 155 | 212 | 151 | 157 | 152 | 54 |
| chr12 | 41422822 | 41423062 | CNTN1 | 0.41 | 666 | 801 | 255 | 661 | 630 | 217 |
| chr12 | 41463738 | 41463858 | CNTN1 | 0.50 | 182 | 214 | 199 | 134 | 206 | 55 |
| chr19 | 10828999 | 10829189 | DNM2 | 0.76 | 9 | 17 | 11 | 4 | 9 | 6 |
| chr19 | 10870390 | 10870510 | DNM2 | 0.59 | 98 | 120 | 114 | 86 | 108 | 44 |
| chr19 | 10883109 | 10883349 | DNM2 | 0.53 | 175 | 171 | 190 | 167 | 161 | 66 |
| chr19 | 10886360 | 10886600 | DNM2 | 0.59 | 139 | 131 | 176 | 143 | 150 | 57 |
| chr19 | 10887782 | 10887902 | DNM2 | 0.58 | 23 | 18 | 42 | 18 | 18 | 9 |
| chr19 | 10893595 | 10893835 | DNM2 | 0.59 | 106 | 113 | 170 | 87 | 82 | 49 |
| chr19 | 10897190 | 10897430 | DNM2 | 0.61 | 212 | 200 | 209 | 201 | 208 | 69 |
| chr19 | 10904343 | 10904583 | DNM2 | 0.58 | 131 | 139 | 159 | 129 | 146 | 39 |
| chr19 | 10906021 | 10906141 | DNM2 | 0.50 | 129 | 217 | 188 | 172 | 150 | 70 |
| chr19 | 10906685 | 10906925 | DNM2 | 0.54 | 94 | 136 | 136 | 133 | 138 | 53 |
| chr19 | 10908004 | 10908244 | DNM2 | 0.53 | 179 | 217 | 140 | 203 | 229 | 129 |
| chr19 | 10909141 | 10909261 | DNM2 | 0.59 | 94 | 115 | 107 | 114 | 177 | 40 |
| chr19 | 10912938 | 10913058 | DNM2 | 0.51 | 82 | 103 | 150 | 113 | 125 | 51 |
| chr19 | 10916557 | 10916677 | DNM2 | 0.57 | 114 | 172 | 147 | 118 | 177 | 49 |
| chr19 | 10922936 | 10923056 | DNM2 | 0.57 | 12 | 18 | 32 | 29 | 31 | 11 |
| chr19 | 10930650 | 10930770 | DNM2 | 0.52 | 75 | 86 | 156 | 78 | 70 | 43 |
| chr19 | 10934459 | 10934579 | DNM2 | 0.62 | 24 | 21 | 28 | 21 | 14 | 3 |
| chr19 | 10935694 | 10935927 | DNM2 | 0.56 | 78 | 79 | 160 | 91 | 63 | 38 |
| chr19 | 10939707 | 10939947 | DNM2 | 0.63 | 125 | 78 | 149 | 88 | 118 | 27 |
| chr19 | 10940748 | 10941108 | DNM2 | 0.68 | 86 | 94 | 132 | 75 | 114 | 61 |
| chr19 | 10941628 | 10941748 | DNM2 | 0.66 | 7 | 9 | 33 | 10 | 19 | 7 |
| chrX | 149761047 | 149761167 | MTM1 | 0.36 | 116 | 187 | 161 | 123 | 266 | 38 |
| chrX | 149764937 | 149765057 | MTM1 | 0.45 | 102 | 107 | 161 | 97 | 135 | 37 |
| chrX | 149767042 | 149767162 | MTM1 | 0.30 | 72 | 115 | 118 | 73 | 147 | 28 |
| chrX | 149783056 | 149783176 | MTM1 | 0.38 | 65 | 92 | 82 | 64 | 162 | 13 |
| chrX | 149787501 | 149787621 | MTM1 | 0.50 | 123 | 149 | 164 | 106 | 228 | 46 |
| chrX | 149807397 | 149807517 | MTM1 | 0.35 | 45 | 73 | 56 | 61 | 98 | 18 |
| chrX | 149809696 | 149809936 | MTM1 | 0.43 | 234 | 315 | 238 | 253 | 499 | 95 |
| chrX | 149814129 | 149814369 | MTM1 | 0.41 | 279 | 353 | 308 | 284 | 554 | 108 |
| chrX | 149818161 | 149818401 | MTM1 | 0.33 | 178 | 198 | 207 | 153 | 280 | 60 |
| chrX | 149826276 | 149826516 | MTM1 | 0.43 | 213 | 221 | 287 | 204 | 375 | 84 |
| chrX | 149828122 | 149828242 | MTM1 | 0.38 | 57 | 98 | 139 | 63 | 156 | 27 |
| chrX | 149828840 | 149828960 | MTM1 | 0.34 | 97 | 122 | 153 | 124 | 215 | 50 |
| chrX | 149831873 | 149832113 | MTM1 | 0.38 | 211 | 269 | 245 | 233 | 362 | 99 |
| chrX | 149839864 | 149840104 | MTM1 | 0.52 | 109 | 120 | 173 | 116 | 249 | 48 |
| chr3 | 9691226 | 9691466 | MTMR14 | 0.71 | 20 | 29 | 25 | 23 | 34 | 9 |
| chr3 | 9695258 | 9695498 | MTMR14 | 0.51 | 413 | 561 | 313 | 433 | 480 | 195 |
| chr3 | 9703944 | 9704064 | MTMR14 | 0.54 | 141 | 193 | 167 | 163 | 177 | 54 |
| chr3 | 9710380 | 9710500 | MTMR14 | 0.49 | 179 | 269 | 182 | 229 | 260 | 69 |
| chr3 | 9711085 | 9711205 | MTMR14 | 0.60 | 42 | 53 | 66 | 34 | 43 | 16 |
| chr3 | 9712672 | 9712906 | MTMR14 | 0.46 | 298 | 334 | 231 | 305 | 298 | 176 |
| chr3 | 9714345 | 9714465 | MTMR14 | 0.53 | 206 | 296 | 185 | 258 | 263 | 101 |
| chr3 | 9718975 | 9719095 | MTMR14 | 0.39 | 46 | 78 | 74 | 54 | 60 | 22 |
| chr3 | 9719644 | 9719764 | MTMR14 | 0.54 | 155 | 211 | 149 | 188 | 163 | 83 |
| chr3 | 9724834 | 9724954 | MTMR14 | 0.46 | 98 | 171 | 99 | 121 | 138 | 43 |
| chr3 | 9726251 | 9726371 | MTMR14 | 0.60 | 31 | 36 | 70 | 37 | 35 | 26 |
| chr3 | 9726549 | 9726669 | MTMR14 | 0.55 | 72 | 141 | 123 | 88 | 96 | 51 |
| chr3 | 9726852 | 9726972 | MTMR14 | 0.53 | 50 | 50 | 74 | 55 | 79 | 20 |
| chr3 | 9729479 | 9729599 | MTMR14 | 0.47 | 26 | 26 | 35 | 28 | 40 | 23 |
| chr3 | 9730348 | 9730468 | MTMR14 | 0.55 | 148 | 247 | 106 | 196 | 230 | 59 |
| chr3 | 9730576 | 9730816 | MTMR14 | 0.61 | 184 | 207 | 150 | 173 | 155 | 80 |
| chr3 | 9731617 | 9731857 | MTMR14 | 0.59 | 117 | 155 | 107 | 119 | 160 | 48 |
| chr3 | 9739352 | 9739592 | MTMR14 | 0.58 | 291 | 273 | 248 | 257 | 273 | 116 |
| chr3 | 9743445 | 9743685 | MTMR14 | 0.59 | 118 | 143 | 108 | 122 | 135 | 50 |
| chr14 | 23882011 | 23882131 | MYH7 | 0.50 | 36 | 50 | 66 | 64 | 45 | 18 |
| chr14 | 23882914 | 23883154 | MYH7 | 0.62 | 159 | 139 | 171 | 181 | 149 | 74 |
| chr14 | 23883203 | 23883323 | MYH7 | 0.63 | 156 | 174 | 154 | 153 | 135 | 75 |
| chr14 | 23884161 | 23884521 | MYH7 | 0.62 | 521 | 632 | 443 | 523 | 513 | 233 |
| chr14 | 23884532 | 23884772 | MYH7 | 0.57 | 320 | 340 | 308 | 333 | 309 | 135 |
| chr14 | 23884819 | 23885059 | MYH7 | 0.62 | 97 | 115 | 170 | 103 | 88 | 89 |
| chr14 | 23885186 | 23885546 | MYH7 | 0.62 | 354 | 339 | 290 | 363 | 423 | 135 |
| chr14 | 23886018 | 23886258 | MYH7 | 0.60 | 189 | 177 | 197 | 213 | 143 | 87 |
| chr14 | 23886324 | 23886564 | MYH7 | 0.61 | 292 | 296 | 200 | 273 | 274 | 144 |
| chr14 | 23886683 | 23886923 | MYH7 | 0.63 | 150 | 266 | 147 | 199 | 223 | 75 |
| chr14 | 23887396 | 23887636 | MYH7 | 0.66 | 97 | 126 | 143 | 86 | 132 | 50 |
| chr14 | 23888324 | 23888564 | MYH7 | 0.59 | 252 | 312 | 309 | 261 | 297 | 126 |
| chr14 | 23888634 | 23888874 | MYH7 | 0.59 | 112 | 119 | 131 | 106 | 101 | 65 |
| chr14 | 23889050 | 23889408 | MYH7 | 0.65 | 169 | 162 | 161 | 176 | 126 | 22 |
| chr14 | 23890151 | 23890271 | MYH7 | 0.55 | 95 | 93 | 99 | 127 | 117 | 38 |
| chr14 | 23891341 | 23891581 | MYH7 | 0.59 | 186 | 199 | 205 | 186 | 185 | 75 |
| chr14 | 23892723 | 23892963 | MYH7 | 0.53 | 521 | 646 | 414 | 529 | 592 | 271 |
| chr14 | 23893056 | 23893416 | MYH7 | 0.53 | 470 | 589 | 392 | 488 | 441 | 245 |
| chr14 | 23893925 | 23894285 | MYH7 | 0.57 | 358 | 457 | 299 | 398 | 360 | 195 |
| chr14 | 23894438 | 23894678 | MYH7 | 0.59 | 175 | 197 | 181 | 200 | 168 | 85 |
| chr14 | 23894845 | 23895085 | MYH7 | 0.51 | 264 | 378 | 270 | 306 | 251 | 103 |
| chr14 | 23895111 | 23895320 | MYH7 | 0.60 | 135 | 177 | 138 | 169 | 113 | 63 |
| chr14 | 23895969 | 23896089 | MYH7 | 0.48 | 68 | 125 | 123 | 100 | 129 | 32 |
| chr14 | 23896422 | 23896542 | MYH7 | 0.54 | 15 | 17 | 25 | 24 | 34 | 11 |
| chr14 | 23896768 | 23897128 | MYH7 | 0.55 | 332 | 391 | 318 | 335 | 296 | 177 |
| chr14 | 23897673 | 23897913 | MYH7 | 0.54 | 272 | 357 | 290 | 360 | 330 | 102 |
| chr14 | 23898118 | 23898358 | MYH7 | 0.55 | 370 | 430 | 359 | 377 | 384 | 165 |
| chr14 | 23898376 | 23898601 | MYH7 | 0.56 | 352 | 436 | 326 | 405 | 425 | 156 |
| chr14 | 23898932 | 23899172 | MYH7 | 0.54 | 294 | 294 | 196 | 274 | 266 | 117 |
| chr14 | 23899760 | 23899880 | MYH7 | 0.54 | 54 | 54 | 94 | 83 | 82 | 33 |
| chr14 | 23900098 | 23900218 | MYH7 | 0.42 | 107 | 180 | 109 | 141 | 148 | 57 |
| chr14 | 23900598 | 23900718 | MYH7 | 0.48 | 53 | 72 | 79 | 64 | 68 | 47 |
| chr14 | 23900779 | 23900899 | MYH7 | 0.62 | 115 | 105 | 85 | 100 | 77 | 70 |
| chr14 | 23900963 | 23901083 | MYH7 | 0.58 | 165 | 199 | 175 | 198 | 253 | 80 |
| chr14 | 23901641 | 23901761 | MYH7 | 0.54 | 148 | 170 | 137 | 141 | 155 | 69 |
| chr14 | 23901805 | 23902045 | MYH7 | 0.59 | 294 | 359 | 250 | 338 | 296 | 110 |
| chr14 | 23902244 | 23902484 | MYH7 | 0.59 | 79 | 104 | 144 | 82 | 73 | 41 |
| chr14 | 23902720 | 23902960 | MYH7 | 0.57 | 300 | 315 | 216 | 296 | 311 | 126 |
| chr19 | 38924431 | 38924551 | RYR1 | 0.62 | 41 | 78 | 59 | 45 | 102 | 17 |
| chr19 | 38931324 | 38931564 | RYR1 | 0.66 | 41 | 51 | 85 | 59 | 50 | 21 |
| chr19 | 38932980 | 38933100 | RYR1 | 0.64 | 8 | 13 | 20 | 5 | 16 | 4 |
| chr19 | 38934174 | 38934294 | RYR1 | 0.66 | 44 | 36 | 59 | 45 | 45 | 19 |
| chr19 | 38934336 | 38934456 | RYR1 | 0.60 | 40 | 37 | 59 | 41 | 47 | 13 |
| chr19 | 38934784 | 38934904 | RYR1 | 0.58 | 42 | 51 | 61 | 43 | 60 | 25 |
| chr19 | 38935210 | 38935330 | RYR1 | 0.64 | 35 | 47 | 38 | 33 | 42 | 21 |
| chr19 | 38937098 | 38937218 | RYR1 | 0.58 | 88 | 95 | 143 | 89 | 104 | 29 |
| chr19 | 38937310 | 38937430 | RYR1 | 0.60 | 29 | 17 | 28 | 23 | 11 | 25 |
| chr19 | 38938952 | 38939192 | RYR1 | 0.66 | 80 | 89 | 128 | 86 | 97 | 41 |
| chr19 | 38939250 | 38939457 | RYR1 | 0.64 | 54 | 67 | 85 | 58 | 64 | 39 |
| chr19 | 38942344 | 38942584 | RYR1 | 0.65 | 127 | 144 | 194 | 128 | 162 | 56 |
| chr19 | 38943436 | 38943676 | RYR1 | 0.66 | 51 | 60 | 58 | 61 | 49 | 15 |
| chr19 | 38945822 | 38946062 | RYR1 | 0.54 | 263 | 365 | 199 | 314 | 318 | 144 |
| chr19 | 38946078 | 38946198 | RYR1 | 0.58 | 121 | 159 | 135 | 161 | 128 | 78 |
| chr19 | 38946211 | 38946451 | RYR1 | 0.58 | 204 | 209 | 215 | 225 | 180 | 89 |
| chr19 | 38948083 | 38948323 | RYR1 | 0.53 | 114 | 150 | 180 | 134 | 138 | 65 |
| chr19 | 38948631 | 38948991 | RYR1 | 0.62 | 515 | 615 | 376 | 552 | 612 | 250 |
| chr19 | 38949761 | 38950001 | RYR1 | 0.62 | 123 | 167 | 196 | 143 | 154 | 59 |
| chr19 | 38951002 | 38951242 | RYR1 | 0.63 | 134 | 168 | 153 | 133 | 119 | 63 |
| chr19 | 38954054 | 38954174 | RYR1 | 0.65 | 53 | 68 | 64 | 55 | 76 | 26 |
| chr19 | 38954378 | 38954498 | RYR1 | 0.56 | 47 | 49 | 53 | 51 | 54 | 18 |
| chr19 | 38955260 | 38955380 | RYR1 | 0.63 | 14 | 15 | 32 | 9 | 22 | 6 |
| chr19 | 38956704 | 38957044 | RYR1 | 0.66 | 59 | 73 | 100 | 55 | 30 | 21 |
| chr19 | 38958230 | 38958470 | RYR1 | 0.61 | 84 | 85 | 117 | 100 | 104 | 34 |
| chr19 | 38959572 | 38959793 | RYR1 | 0.59 | 197 | 259 | 251 | 218 | 251 | 91 |
| chr19 | 38959939 | 38960168 | RYR1 | 0.60 | 110 | 136 | 151 | 85 | 102 | 49 |
| chr19 | 38963973 | 38964453 | RYR1 | 0.69 | 34 | 25 | 66 | 23 | 37 | 16 |
| chr19 | 38965903 | 38966143 | RYR1 | 0.61 | 81 | 94 | 109 | 86 | 100 | 40 |
| chr19 | 38968309 | 38968549 | RYR1 | 0.62 | 23 | 26 | 64 | 31 | 37 | 20 |
| chr19 | 38969037 | 38969277 | RYR1 | 0.56 | 136 | 154 | 157 | 101 | 137 | 59 |
| chr19 | 38973649 | 38973769 | RYR1 | 0.55 | 112 | 145 | 161 | 136 | 141 | 61 |
| chr19 | 38973922 | 38974162 | RYR1 | 0.67 | 20 | 16 | 28 | 21 | 20 | 13 |
| chr19 | 38976175 | 38976895 | RYR1 | 0.65 | 630 | 663 | 631 | 671 | 752 | 279 |
| chr19 | 38979764 | 38979884 | RYR1 | 0.50 | 89 | 95 | 143 | 93 | 86 | 47 |
| chr19 | 38980006 | 38980126 | RYR1 | 0.56 | 149 | 220 | 209 | 141 | 170 | 84 |
| chr19 | 38980695 | 38980935 | RYR1 | 0.61 | 133 | 145 | 201 | 130 | 140 | 65 |
| chr19 | 38981256 | 38981376 | RYR1 | 0.44 | 176 | 231 | 120 | 195 | 197 | 72 |
| chr19 | 38983101 | 38983322 | RYR1 | 0.59 | 127 | 190 | 136 | 157 | 157 | 36 |
| chr19 | 38984948 | 38985308 | RYR1 | 0.65 | 225 | 254 | 235 | 223 | 236 | 94 |
| chr19 | 38986851 | 38986971 | RYR1 | 0.57 | 88 | 128 | 107 | 102 | 116 | 52 |
| chr19 | 38986994 | 38987234 | RYR1 | 0.64 | 113 | 168 | 175 | 161 | 176 | 57 |
| chr19 | 38987486 | 38987606 | RYR1 | 0.63 | 31 | 33 | 80 | 33 | 24 | 18 |
| chr19 | 38989695 | 38989935 | RYR1 | 0.61 | 136 | 142 | 186 | 148 | 179 | 67 |
| chr19 | 38990247 | 38990487 | RYR1 | 0.69 | 34 | 47 | 61 | 50 | 38 | 7 |
| chr19 | 38990541 | 38990661 | RYR1 | 0.59 | 30 | 17 | 47 | 39 | 35 | 10 |
| chr19 | 38991185 | 38991665 | RYR1 | 0.65 | 132 | 145 | 230 | 120 | 143 | 42 |
| chr19 | 38993136 | 38993376 | RYR1 | 0.64 | 28 | 62 | 117 | 50 | 96 | 15 |
| chr19 | 38993504 | 38993624 | RYR1 | 0.63 | 64 | 85 | 125 | 80 | 127 | 31 |
| chr19 | 38994809 | 38995049 | RYR1 | 0.59 | 67 | 50 | 81 | 63 | 76 | 33 |
| chr19 | 38995349 | 38995589 | RYR1 | 0.58 | 224 | 308 | 244 | 276 | 247 | 128 |
| chr19 | 38995621 | 38995741 | RYR1 | 0.56 | 151 | 199 | 172 | 198 | 204 | 84 |
| chr19 | 38995933 | 38996053 | RYR1 | 0.54 | 57 | 67 | 58 | 29 | 75 | 35 |
| chr19 | 38996395 | 38996635 | RYR1 | 0.59 | 65 | 82 | 100 | 84 | 125 | 58 |
| chr19 | 38996919 | 38997039 | RYR1 | 0.59 | 91 | 129 | 99 | 101 | 121 | 45 |
| chr19 | 38997088 | 38997208 | RYR1 | 0.59 | 50 | 62 | 51 | 50 | 36 | 29 |
| chr19 | 38997410 | 38997650 | RYR1 | 0.63 | 104 | 124 | 55 | 99 | 100 | 39 |
| chr19 | 38998349 | 38998469 | RYR1 | 0.54 | 130 | 170 | 151 | 149 | 215 | 50 |
| chr19 | 39001111 | 39001231 | RYR1 | 0.52 | 188 | 248 | 175 | 208 | 227 | 87 |
| chr19 | 39001240 | 39001480 | RYR1 | 0.57 | 312 | 394 | 281 | 390 | 413 | 162 |
| chr19 | 39002165 | 39002285 | RYR1 | 0.53 | 207 | 236 | 192 | 203 | 249 | 88 |
| chr19 | 39002681 | 39002801 | RYR1 | 0.67 | 69 | 50 | 82 | 61 | 60 | 42 |
| chr19 | 39002823 | 39003183 | RYR1 | 0.62 | 241 | 275 | 345 | 236 | 249 | 121 |
| chr19 | 39005646 | 39005766 | RYR1 | 0.55 | 64 | 84 | 117 | 80 | 97 | 32 |
| chr19 | 39006671 | 39006911 | RYR1 | 0.66 | 15 | 19 | 21 | 17 | 12 | 4 |
| chr19 | 39007984 | 39008344 | RYR1 | 0.66 | 82 | 112 | 194 | 116 | 155 | 46 |
| chr19 | 39009793 | 39010153 | RYR1 | 0.68 | 74 | 57 | 98 | 86 | 91 | 29 |
| chr19 | 39013651 | 39013771 | RYR1 | 0.65 | 20 | 20 | 21 | 12 | 8 | 13 |
| chr19 | 39013842 | 39013962 | RYR1 | 0.50 | 40 | 51 | 98 | 58 | 69 | 20 |
| chr19 | 39014501 | 39014621 | RYR1 | 0.60 | 32 | 78 | 69 | 29 | 72 | 10 |
| chr19 | 39015953 | 39016176 | RYR1 | 0.64 | 88 | 105 | 119 | 98 | 128 | 38 |
| chr19 | 39017602 | 39017722 | RYR1 | 0.52 | 47 | 88 | 58 | 57 | 67 | 16 |
| chr19 | 39018235 | 39018475 | RYR1 | 0.66 | 49 | 62 | 60 | 43 | 89 | 21 |
| chr19 | 39018941 | 39019061 | RYR1 | 0.60 | 106 | 132 | 113 | 133 | 168 | 66 |
| chr19 | 39019226 | 39019346 | RYR1 | 0.54 | 73 | 70 | 105 | 71 | 80 | 41 |
| chr19 | 39019477 | 39019597 | RYR1 | 0.55 | 56 | 51 | 42 | 53 | 34 | 30 |
| chr19 | 39019638 | 39019758 | RYR1 | 0.65 | 52 | 76 | 68 | 74 | 73 | 22 |
| chr19 | 39023095 | 39023215 | RYR1 | 0.55 | 69 | 116 | 101 | 87 | 115 | 44 |
| chr19 | 39023283 | 39023403 | RYR1 | 0.61 | 58 | 73 | 81 | 46 | 43 | 33 |
| chr19 | 39025349 | 39025469 | RYR1 | 0.61 | 45 | 33 | 65 | 47 | 54 | 22 |
| chr19 | 39025760 | 39025880 | RYR1 | 0.56 | 84 | 74 | 76 | 71 | 66 | 69 |
| chr19 | 39025933 | 39026053 | RYR1 | 0.50 | 180 | 181 | 209 | 215 | 260 | 67 |
| chr19 | 39026613 | 39026733 | RYR1 | 0.55 | 26 | 38 | 63 | 43 | 45 | 10 |
| chr19 | 39027355 | 39027475 | RYR1 | 0.64 | 8 | 9 | 33 | 10 | 19 | 9 |
| chr19 | 39028499 | 39028619 | RYR1 | 0.53 | 23 | 38 | 69 | 31 | 28 | 18 |
| chr19 | 39033970 | 39034090 | RYR1 | 0.50 | 210 | 263 | 203 | 244 | 223 | 98 |
| chr19 | 39034115 | 39034355 | RYR1 | 0.62 | 243 | 294 | 212 | 214 | 242 | 133 |
| chr19 | 39034402 | 39034522 | RYR1 | 0.64 | 77 | 81 | 72 | 60 | 85 | 24 |
| chr19 | 39037065 | 39037185 | RYR1 | 0.55 | 91 | 109 | 123 | 115 | 112 | 34 |
| chr19 | 39038846 | 39039086 | RYR1 | 0.57 | 267 | 322 | 294 | 298 | 288 | 112 |
| chr19 | 39051743 | 39052103 | RYR1 | 0.61 | 86 | 119 | 181 | 109 | 101 | 41 |
| chr19 | 39055553 | 39055750 | RYR1 | 0.64 | 11 | 27 | 38 | 13 | 18 | 4 |
| chr19 | 39056031 | 39056462 | RYR1 | 0.73 | 6 | 4 | 9 | 2 | 8 | 4 |
| chr19 | 39057528 | 39057648 | RYR1 | 0.66 | 23 | 18 | 22 | 19 | 25 | 6 |
| chr19 | 39058364 | 39058604 | RYR1 | 0.61 | 87 | 105 | 93 | 102 | 96 | 42 |
| chr19 | 39061229 | 39061349 | RYR1 | 0.47 | 230 | 331 | 203 | 285 | 282 | 94 |
| chr19 | 39062604 | 39062929 | RYR1 | 0.59 | 159 | 172 | 143 | 165 | 152 | 58 |
| chr19 | 39063761 | 39064001 | RYR1 | 0.62 | 36 | 62 | 65 | 75 | 50 | 18 |
| chr19 | 39066519 | 39066639 | RYR1 | 0.50 | 162 | 224 | 178 | 209 | 256 | 85 |
| chr19 | 39068502 | 39068742 | RYR1 | 0.62 | 211 | 198 | 222 | 198 | 190 | 64 |
| chr19 | 39068753 | 39068873 | RYR1 | 0.60 | 60 | 46 | 67 | 61 | 58 | 27 |
| chr19 | 39070574 | 39070814 | RYR1 | 0.61 | 229 | 294 | 257 | 238 | 260 | 121 |
| chr19 | 39070956 | 39071162 | RYR1 | 0.61 | 121 | 147 | 178 | 163 | 157 | 66 |
| chr19 | 39075560 | 39075773 | RYR1 | 0.60 | 84 | 111 | 139 | 95 | 116 | 36 |
| chr19 | 39076549 | 39076669 | RYR1 | 0.56 | 65 | 69 | 77 | 55 | 44 | 21 |
| chr19 | 39076720 | 39076840 | RYR1 | 0.53 | 80 | 90 | 124 | 90 | 86 | 35 |
| chr19 | 39077130 | 39077250 | RYR1 | 0.50 | 73 | 96 | 94 | 74 | 85 | 35 |
| chr19 | 39077952 | 39078072 | RYR1 | 0.50 | 126 | 214 | 124 | 206 | 199 | 52 |
| chr1 | 26126859 | 26126979 | SEPN1 | 0.82 | 0 | 3 | 1 | 1 | 0 | 1 |
| chr1 | 26127472 | 26127712 | SEPN1 | 0.59 | 42 | 37 | 67 | 29 | 33 | 16 |
| chr1 | 26131579 | 26131819 | SEPN1 | 0.60 | 136 | 112 | 109 | 140 | 132 | 48 |
| chr1 | 26135055 | 26135295 | SEPN1 | 0.66 | 38 | 42 | 48 | 42 | 61 | 30 |
| chr1 | 26135458 | 26135679 | SEPN1 | 0.65 | 110 | 113 | 147 | 130 | 107 | 26 |
| chr1 | 26136122 | 26136362 | SEPN1 | 0.63 | 190 | 244 | 177 | 179 | 245 | 93 |
| chr1 | 26137925 | 26138045 | SEPN1 | 0.60 | 58 | 65 | 77 | 58 | 57 | 16 |
| chr1 | 26138155 | 26138395 | SEPN1 | 0.64 | 25 | 20 | 27 | 26 | 21 | 20 |
| chr1 | 26139170 | 26139290 | SEPN1 | 0.63 | 29 | 22 | 35 | 19 | 29 | 7 |
| chr1 | 26140367 | 26140487 | SEPN1 | 0.60 | 34 | 40 | 68 | 40 | 33 | 22 |
| chr1 | 26140558 | 26140678 | SEPN1 | 0.60 | 41 | 45 | 76 | 35 | 44 | 16 |
| chr1 | 26142003 | 26142243 | SEPN1 | 0.60 | 152 | 151 | 178 | 162 | 134 | 62 |
| chr19 | 55644245 | 55644365 | TNNT1 | 0.64 | 25 | 32 | 15 | 29 | 28 | 11 |
| chr19 | 55645214 | 55645334 | TNNT1 | 0.60 | 43 | 37 | 63 | 49 | 47 | 18 |
| chr19 | 55645382 | 55645583 | TNNT1 | 0.64 | 45 | 40 | 83 | 42 | 56 | 22 |
| chr19 | 55648465 | 55648585 | TNNT1 | 0.60 | 32 | 51 | 101 | 41 | 67 | 13 |
| chr19 | 55649325 | 55649445 | TNNT1 | 0.55 | 93 | 139 | 124 | 106 | 153 | 54 |
| chr19 | 55652229 | 55652349 | TNNT1 | 0.62 | 21 | 38 | 20 | 31 | 33 | 18 |
| chr19 | 55652491 | 55652731 | TNNT1 | 0.61 | 157 | 213 | 151 | 177 | 172 | 67 |
| chr19 | 55653201 | 55653321 | TNNT1 | 0.59 | 20 | 23 | 23 | 19 | 25 | 7 |
| chr19 | 55656862 | 55656982 | TNNT1 | 0.47 | 51 | 64 | 72 | 88 | 77 | 18 |
| chr19 | 55657757 | 55657877 | TNNT1 | 0.73 | 3 | 2 | 8 | 2 | 1 | 0 |
| chr19 | 55658001 | 55658121 | TNNT1 | 0.69 | 1 | 5 | 14 | 5 | 4 | 4 |
| chr19 | 55658322 | 55658442 | TNNT1 | 0.68 | 36 | 54 | 60 | 30 | 44 | 16 |
| chr19 | 55658449 | 55658569 | TNNT1 | 0.63 | 42 | 73 | 80 | 44 | 67 | 16 |
| chr9 | 35682058 | 35682178 | TPM2 | 0.61 | 26 | 49 | 79 | 54 | 63 | 19 |
| chr9 | 35683136 | 35683256 | TPM2 | 0.58 | 8 | 10 | 31 | 10 | 11 | 6 |
| chr9 | 35684217 | 35684337 | TPM2 | 0.50 | 92 | 110 | 59 | 91 | 94 | 50 |
| chr9 | 35684455 | 35684575 | TPM2 | 0.47 | 111 | 127 | 140 | 94 | 140 | 74 |
| chr9 | 35684706 | 35684826 | TPM2 | 0.58 | 81 | 124 | 157 | 76 | 119 | 49 |
| chr9 | 35685038 | 35685158 | TPM2 | 0.64 | 68 | 66 | 91 | 47 | 54 | 33 |
| chr9 | 35685240 | 35685360 | TPM2 | 0.62 | 155 | 111 | 99 | 116 | 80 | 71 |
| chr9 | 35685369 | 35685830 | TPM2 | 0.60 | 410 | 532 | 372 | 448 | 483 | 187 |
| chr9 | 35689085 | 35689325 | TPM2 | 0.61 | 119 | 137 | 136 | 97 | 123 | 53 |
| chr9 | 35689697 | 35689817 | TPM2 | 0.61 | 164 | 241 | 161 | 217 | 291 | 69 |
| chr1 | 154130095 | 154130215 | TPM3 | 0.51 | 119 | 156 | 149 | 126 | 137 | 39 |
| chr1 | 154131424 | 154131544 | TPM3 | 0.55 | 128 | 143 | 115 | 130 | 125 | 34 |
| chr1 | 154140354 | 154140474 | TPM3 | 0.45 | 68 | 120 | 73 | 73 | 84 | 29 |
| chr1 | 154141759 | 154141879 | TPM3 | 0.50 | 129 | 170 | 176 | 176 | 128 | 53 |
| chr1 | 154142850 | 154142970 | TPM3 | 0.48 | 226 | 294 | 198 | 210 | 265 | 97 |
| chr1 | 154143095 | 154143215 | TPM3 | 0.34 | 118 | 140 | 102 | 129 | 133 | 72 |
| chr1 | 154143866 | 154143986 | TPM3 | 0.47 | 179 | 232 | 145 | 162 | 218 | 70 |
| chr1 | 154144482 | 154144602 | TPM3 | 0.50 | 74 | 139 | 100 | 97 | 90 | 32 |
| chr1 | 154145358 | 154145478 | TPM3 | 0.50 | 192 | 215 | 134 | 167 | 141 | 112 |
| chr1 | 154145498 | 154145738 | TPM3 | 0.44 | 440 | 569 | 267 | 433 | 428 | 227 |
| chr1 | 154148537 | 154148777 | TPM3 | 0.51 | 330 | 459 | 293 | 393 | 335 | 158 |
| chr1 | 154155409 | 154155649 | TPM3 | 0.65 | 197 | 190 | 261 | 166 | 239 | 101 |
| chr1 | 154163604 | 154163844 | TPM3 | 0.46 | 300 | 373 | 305 | 307 | 228 | 113 |
| chr1 | 154164315 | 154164555 | TPM3 | 0.49 | 340 | 475 | 307 | 329 | 388 | 166 |
| chr2 | 179610298 | 179616778 | TTN | 0.38 | 12056 | 13619 | 6328 | 11786 | 9969 | 5425 |
| chr2 | 179617818 | 179617938 | TTN | 0.29 | 77 | 142 | 109 | 112 | 89 | 43 |
| chr2 | 179623684 | 179623924 | TTN | 0.41 | 443 | 559 | 262 | 441 | 458 | 175 |
| chr2 | 179628846 | 179629086 | TTN | 0.44 | 343 | 420 | 222 | 313 | 277 | 136 |
| chr2 | 179629215 | 179629575 | TTN | 0.49 | 323 | 344 | 152 | 348 | 247 | 141 |
| chr2 | 179631103 | 179631343 | TTN | 0.42 | 462 | 566 | 292 | 503 | 442 | 196 |
| chr2 | 179632448 | 179632688 | TTN | 0.42 | 601 | 717 | 274 | 579 | 498 | 226 |
| chr2 | 179632691 | 179632910 | TTN | 0.40 | 408 | 446 | 279 | 407 | 352 | 161 |
| chr2 | 179633349 | 179633709 | TTN | 0.39 | 384 | 455 | 450 | 376 | 331 | 155 |
| chr2 | 179634475 | 179634715 | TTN | 0.39 | 256 | 265 | 162 | 257 | 200 | 132 |
| chr2 | 179634736 | 179635450 | TTN | 0.43 | 1508 | 1744 | 739 | 1565 | 1327 | 654 |
| chr2 | 179635887 | 179636247 | TTN | 0.43 | 501 | 538 | 352 | 440 | 445 | 206 |
| chr2 | 179637785 | 179638500 | TTN | 0.36 | 1088 | 1168 | 631 | 1058 | 872 | 487 |
| chr2 | 179638520 | 179638880 | TTN | 0.42 | 881 | 896 | 449 | 879 | 748 | 341 |
| chr2 | 179638886 | 179639246 | TTN | 0.39 | 697 | 841 | 417 | 710 | 631 | 296 |
| chr2 | 179639608 | 179639968 | TTN | 0.39 | 614 | 723 | 429 | 595 | 558 | 287 |
| chr2 | 179640029 | 179641829 | TTN | 0.45 | 3590 | 4263 | 1952 | 3741 | 3221 | 1664 |
| chr2 | 179641839 | 179642079 | TTN | 0.37 | 517 | 563 | 255 | 498 | 444 | 207 |
| chr2 | 179642108 | 179642348 | TTN | 0.34 | 358 | 397 | 178 | 356 | 246 | 154 |
| chr2 | 179642386 | 179642746 | TTN | 0.43 | 438 | 568 | 332 | 412 | 411 | 205 |
| chr2 | 179643542 | 179643902 | TTN | 0.37 | 488 | 571 | 290 | 518 | 375 | 200 |
| chr2 | 179643952 | 179644192 | TTN | 0.33 | 326 | 348 | 205 | 266 | 200 | 105 |
| chr2 | 179644672 | 179644792 | TTN | 0.32 | 237 | 285 | 164 | 223 | 243 | 87 |
| chr2 | 179644819 | 179644939 | TTN | 0.40 | 262 | 322 | 159 | 266 | 253 | 82 |
| chr2 | 179645798 | 179646038 | TTN | 0.36 | 246 | 289 | 275 | 276 | 244 | 112 |
| chr2 | 179646926 | 179647166 | TTN | 0.49 | 288 | 361 | 189 | 301 | 301 | 126 |
| chr2 | 179647237 | 179647357 | TTN | 0.33 | 127 | 141 | 82 | 126 | 95 | 76 |
| chr2 | 179647481 | 179647841 | TTN | 0.46 | 442 | 463 | 272 | 414 | 320 | 163 |
| chr2 | 179648419 | 179648539 | TTN | 0.37 | 187 | 239 | 132 | 234 | 239 | 59 |
| chr2 | 179648757 | 179649117 | TTN | 0.50 | 506 | 604 | 381 | 537 | 491 | 216 |
| chr2 | 179650287 | 179650527 | TTN | 0.39 | 384 | 425 | 310 | 443 | 360 | 170 |
| chr2 | 179650541 | 179650901 | TTN | 0.49 | 437 | 428 | 334 | 392 | 353 | 154 |
| chr2 | 179654035 | 179654275 | TTN | 0.37 | 386 | 462 | 268 | 448 | 388 | 149 |
| chr2 | 179654653 | 179654893 | TTN | 0.32 | 384 | 432 | 305 | 356 | 375 | 144 |
| chr2 | 179655383 | 179655623 | TTN | 0.39 | 412 | 504 | 298 | 491 | 407 | 158 |
| chr2 | 179656741 | 179656981 | TTN | 0.30 | 150 | 172 | 115 | 147 | 144 | 62 |
| chr2 | 179658079 | 179658319 | TTN | 0.39 | 328 | 468 | 323 | 403 | 373 | 149 |
| chr2 | 179659081 | 179659321 | TTN | 0.50 | 153 | 220 | 166 | 168 | 180 | 82 |
| chr2 | 179659633 | 179659993 | TTN | 0.56 | 243 | 292 | 290 | 272 | 262 | 117 |
| chr2 | 179664162 | 179664515 | TTN | 0.52 | 284 | 275 | 189 | 259 | 254 | 120 |
| chr2 | 179664534 | 179664654 | TTN | 0.38 | 167 | 204 | 82 | 189 | 182 | 74 |
| chr2 | 179665085 | 179665445 | TTN | 0.51 | 696 | 859 | 397 | 794 | 756 | 342 |
| chr2 | 179666846 | 179667086 | TTN | 0.54 | 308 | 364 | 252 | 380 | 301 | 138 |
| chr2 | 179669263 | 179669383 | TTN | 0.50 | 99 | 138 | 110 | 114 | 130 | 54 |
