# Supplementary material for: An Integrated Diagnosis Strategy for Congenital Myopathies
Source: PLoS One. 2013 Jun 24;8(6):e67527. doi: 10.1371/journal.pone.0067527 (PMC3691193; doi:10.1371/journal.pone.0067527)
Supplement: Table S5 — Web resources. (DOCX) [file pone.0067527.s007.docx]

**Table S5:** Web resources

| **Name** | **Available** | **Accessed** |
| --- | --- | --- |
| 1000 genomes | <http://www.1000genomes.org/> | Nov 2012 |
| Database of Single Nucleotide Polymorphisms (dbSNP Build ID: 134) | <http://www.ncbi.nlm.nih.gov/SNP/> | Nov 2012 |
| Ensembl60 | <http://www.ensembl.org/index.html> | Nov 2012 |
| Exome Variant Server, NHLBI Exome Sequencing Project (ESP) | <http://evs.gs.washington.edu/EVS/> | Nov 2012 |
| Human Splicing Finder | <http://www.umd.be/HSF/> | Nov 2012 |
| Online Mendelian Inheritance in Man (OMIM) | <http://www.omim.org/> | Nov 2012 |
| NNsplice - **Berkeley Drosophila Genome Project** | <http://www.fruitfly.org/seq_tools/splice.html> | Nov 2012 |
| PolyPhen-2 | <http://genetics.bwh.harvard.edu/pph2/> | Nov 2012 |
| SIFT | <http://sift.jcvi.org/> | Nov 2012 |
